# Global variation in bacterial strains that cause tuberculosis disease: a systematic review and meta-analysis

**DOI:** 10.1186/s12916-018-1180-x

**Published:** 2018-10-30

**Authors:** Kirsten E Wiens, Lauren P Woyczynski, Jorge R Ledesma, Jennifer M Ross, Roberto Zenteno-Cuevas, Amador Goodridge, Irfan Ullah, Barun Mathema, Joel Fleury Djoba Siawaya, Molly H Biehl, Sarah E Ray, Natalia V Bhattacharjee, Nathaniel J Henry, Robert C Reiner, Hmwe H Kyu, Christopher J L Murray, Simon I Hay

**Affiliations:** 1 Institute for Health Metrics and Evaluation, University of Washington, 2301 5th Ave, Suite 600, Seattle, WA 98121 USA; 2 Departments of Global Health and Medicine, University of Washington, Seattle, WA USA; 3 Public Health Institute, University of Veracruz, Veracruz, Mexico; 4 Tuberculosis Biomarker Research Unit, Instituto de Investigaciones Científicas y Servicios de Alta Tecnología (INDICASAT-AIP), City of Knowledge, Panama, Panama; 5 Gomal Centre of Biochemistry and Biotechnology, Gomal University, Dera Ismail Khan, Khyber Pakhtunkhwa Pakistan; 6 Programmatic Management of Drug-Resistant TB Unit, BSL-II TB Culture Laboratory, Mufti Mehmood Memorial Teaching Hospital, Dera Ismail Khan, Khyber Pakhtunkhwa Pakistan; 7 Department of Epidemiology, Mailman School of Public Health, Columbia University, New York, NY USA; 8 Unité de Recherche et de Diagnostics Spécialisés, Laboratoire National de Santé Publique, Libreville, Gabon; 9 Centre Hospitalier Universitaire Mère-Enfant Fondation Jeanne EBORI, Libreville, Gabon

**Keywords:** Tuberculosis, *Mycobacterium tuberculosis*, Genotype, Genetic variation, Epidemiology, Molecular epidemiology

## Abstract

**Background:**

The host, microbial, and environmental factors that contribute to variation in tuberculosis (TB) disease are incompletely understood. Accumulating evidence suggests that one driver of geographic variation in TB disease is the local ecology of mycobacterial genotypes or strains, and there is a need for a comprehensive and systematic synthesis of these data. The objectives of this study were to (1) map the global distribution of genotypes that cause TB disease and (2) examine whether any epidemiologically relevant clinical characteristics were associated with those genotypes.

**Methods:**

We performed a systematic review of PubMed and Scopus to create a comprehensive dataset of human TB molecular epidemiology studies that used representative sampling techniques. The methods were developed according to the Preferred Reporting Items for Systematic Reviews and Meta-Analyses (PRISMA). We extracted and synthesized data from studies that reported prevalence of bacterial genotypes and from studies that reported clinical characteristics associated with those genotypes.

**Results:**

The results of this study are twofold. First, we identified 206 studies for inclusion in the study, representing over 200,000 bacterial isolates collected over 27 years in 85 countries. We mapped the genotypes and found that, consistent with previously published maps, Euro-American lineage 4 and East Asian lineage 2 strains are widespread, and West African lineages 5 and 6 strains are geographically restricted. Second, 30 studies also reported transmission chains and 4 reported treatment failure associated with genotypes. We performed a meta-analysis and found substantial heterogeneity across studies. However, based on the data available, we found that lineage 2 strains may be associated with increased risk of transmission chains, while lineages 5 and 6 strains may be associated with reduced risk, compared with lineage 4 strains.

**Conclusions:**

This study provides the most comprehensive systematic analysis of the evidence for diversity in bacterial strains that cause TB disease. The results show both geographic and epidemiological differences between strains, which could inform our understanding of the global burden of TB. Our findings also highlight the challenges of collecting the clinical data required to inform TB diagnosis and treatment. We urge future national TB programs and research efforts to prioritize and reinforce clinical data collection in study designs and results dissemination.

**Electronic supplementary material:**

The online version of this article (10.1186/s12916-018-1180-x) contains supplementary material, which is available to authorized users.

## Background

Tuberculosis (TB) is found in every population of the world today and kills 1.1–1.6 million people globally each year [1]. There is also significant geographic variation in the prevalence, incidence, and mortality of TB [1]. The factors that contribute to individual and geographic variation in TB infection and disease are incompletely understood. An intact immune response is required to prevent infection and progression to active disease as conditions that weaken the immune system are strongly associated with TB, including HIV co-infection, type II diabetes mellitus, undernutrition, and immunosuppressive medications such as anti-tumor necrosis factor (TNF) therapy [2]. Environmental factors likely also play a role in infection and disease progression, including population density, indoor and outdoor air pollution, and health care quality and access [2]. However, these risk factors are insufficient to explain the current burden of TB [3].

An additional driver of variation may be human and bacterial genetic variation [4]. There are human genetic polymorphisms associated with susceptibility to latent TB infection and progression to active disease [5], as well as polymorphisms in the *Mycobacterium tuberculosis* complex (MTBC) associated with the ability to cause disease [6] and with transmissibility [7]. The host-pathogen relationship in TB is sympatric [8], i.e., the host and pathogen tend to share a common ancestral geographic origin [8]. When patients are infected with an allopatric strain or a strain that originates from a different geographic origin than the patient, they may be at risk for greater pulmonary impairment [9]. Similarly, there is evidence for associations between human leukocyte antigen (HLA) type and susceptibility to TB disease caused by particular MTBC strains [10, 11]. However, there is considerable variation in studies that test for associations between MTBC genotypes and clinical characteristics [12, 13].

A better understanding of MTBC molecular epidemiology could improve our ability to treat and control TB. Genetic data are already being used by epidemiologists as tools for outbreak investigations to identify sources of mycobacterial infection [14] and as tools in surveillance to identify the strains most likely to spread rapidly through new human populations [3]. Additionally, understanding the risk factors associated with MTBC genetic data could help direct the development of biomarker-based diagnostic tests to identify patients early that are infected with strains associated with higher risk of treatment failure, relapse, drug resistance, or death [15]. Finally, there is accumulating evidence for variation in the immune response to distinct MTBC strains [16–21]. Therefore, understanding the global variation in MTBC strains will be important as new vaccines, biomarkers, and host-directed therapies are developed [13].

The objective of this study was to systematically synthesize all available information on MTBC genotypes in order to (1) map the global distribution of genotypes that cause TB disease and (2) determine whether any epidemiologically relevant clinical characteristics were associated with those genotypes. Previous systematic reviews that mapped MTBC genotype distribution focused on MTBC Beijing family strains and their association with drug resistance [22, 23]. We expanded on this previous work by considering data for all MTBC lineages, making this the most comprehensive synthesis of MTBC genotypes that has been conducted to date.

## Methods

The methods for this systematic review, including literature search, inclusion criteria, and analysis, were developed according to the Preferred Reporting Items for Systematic Reviews and Meta-Analyses (PRISMA) [24, 25].

### Information sources and search strategy

We identified studies by systematically searching PubMed and Scopus. The first search was run on June 8, 2017, and the final search was run on November 13, 2017. Articles identified in these searches were supplemented by six published studies in Papua New Guinea, India, Botswana, Nepal, Ethiopia, and Kenya, and two unpublished studies in Mexico and Panama, which we were directed to by manually checking the reference lists of studies and by reviewing the conference abstracts for the 48th Union World Conference on Lung Health. Complete details of search strings and dates searched are found in Additional file 1.

### Eligibility criteria

#### Types of studies

In order to minimize sampling bias in the analysis, we restricted our analysis to human TB molecular epidemiology studies that used either probability sampling methods, such as random or cluster-based sampling, or that collected samples from all reported or all new TB cases in the study location and time period. For the majority of studies “all TB cases” included culture-positive TB cases only. For studies that used GeneXpert remnants for DNA collection, this included microscopy-positive TB cases. We excluded studies of sub-populations that may over- or underrepresent particular genotypes, such as studies restricted to hospital workers, prisoners, HIV-infected individuals, children, homeless individuals, immigrants, individuals living in slums, military personnel, individuals with drug-resistant strains, relapse or re-infection cases, or extrapulmonary TB cases. In addition, we excluded outbreak investigations, case studies, review articles, and studies not available in English or Spanish. When multiple studies used the same data, we included the study that provided the most detailed genotyping data and/or the most detailed corresponding clinical data. For the global mapping analysis, we excluded studies that only tested or reported data for one lineage. These latter studies were considered for the clinical characteristics analysis. We did not apply publication date restrictions.

#### Types of genotyping methods

We included studies that reported genotyping results by geographic location, year, and sampling method and that met the eligibility criteria described above. We considered genotypes determined by whole-genome sequencing (WGS), large sequence polymorphism (LSP) as determined by polymerase chain reaction (PCR), spacer oligonucleotide typing (spoligotyping), and multi-locus variable number of tandem repeats (VNTR) analysis (MLVA).

#### Types of clinical characteristics

In a secondary step, we screened all studies that met our initial inclusion criteria for studies that also reported clinical characteristics associated with genotypes, including transmission chains, progression to active TB, treatment failure, duration of symptoms, relapse or retreatment, severity of pulmonary lesions, and extrapulmonary TB. For further analysis, we focused on the characteristics with clear case definitions and sufficient data available for meta-analysis, which included treatment failure and transmission chains. Treatment failure was defined as a TB case that had a positive sputum culture and/or smear at 5–8 months following the start of TB treatment. Transmission chains were inferred by genetic clusters, which were defined as two or more identical genotype patterns identified in the same study location and time period [26]. Genetic clustering appears not to be a perfect measure of transmission chains since it can be impacted by various factors including social mixing, immigration, age structure, and underlying TB incidence [27, 28]. However, we decided that it was an important measure to include because (1) it has been an important tool for TB surveillance [29–31] and (2) it currently has sufficient published data available for global analysis.

### Data collection process and data items

#### Study screening and selection

Articles were reviewed for eligibility first by screening the titles and abstracts and then by reviewing the full texts in an unblinded standardized manner. One reviewer screened the titles and abstracts, and the selected articles were divided between three reviewers to screen and extract using a standardized data extraction form. When there was uncertainty about the eligibility, reviewers arrived at a decision by consensus.

#### Genotype data extraction

Information that was extracted from each study included (1) page number and table or figure the data was extracted from, (2) underlying study design [cohort or cross-sectional], (3) sampling approach [all cases, all new cases, cluster-based sample, or random sample], (4) geographic region and year(s) the sample represented, (5) genotyping method [spoligotyping, MLVA typing, PCR, or WGS], and (6) total count of each genotype identified in the sample. Additional file 2 contains the screening sheet detailing all studies reviewed, reason(s) for exclusion, and a log of any follow-up. Additional file 3 contains all raw genotyping data extracted and corresponding study meta-data. The original extraction sheet is available upon request.

#### Clinical characteristic data extraction

All studies that reported transmission chains or genetic clustering as defined above were included in a second extraction sheet. The following additional data were extracted in this sheet: (1) the total count of each genotype, (2) total count of each genotype that was part of a genetic cluster, and (3) potential confounders, when available (including the proportion of HIV co-infection and drug resistance in the sample, the mean age of the participants, and the proportion of participants that were male, had previously been diagnosed with TB, had extrapulmonary TB, or were immigrants). Additional file 4 contains raw genetic clustering data extracted and corresponding study meta-data.

### Synthesis and analysis

#### Classification system for genotypes

In this study, we defined “strains” based on the seven phylogenetic lineages identified by S. Gagneaux and colleagues [32]. We included data on animal lineage strains isolated from human TB cases, which included *Mycobacterium bovis*, *Mycobacterium pinnipedii*, *Mycobacterium caprae*, *Mycobacterium origys*, and *Mycobacterium microti* strains. Some studies reported “other,” “unknown,” “undefined,” “unclear,” or “uncommon” genotypes, which we labeled as “unknown lineages.” For each study, we extracted data at the most detailed genotype level available. When spoligotype octal codes were provided, we determined phylogenetic lineage using the central Bayesian network (CBN) method implemented in Run TB-Lineage [33, 34]. When spoligotype clade or family was provided, we used SITVIT Web and Run TB-Lineage to determine phylogenetic lineage [35]. When MLVA family was provided, we determined phylogenetic lineage using MIRU-VNTR*plus* [36, 37]. For Ethiopian MLVA families, we assigned strains to lineage 4 or lineage 7 using genetic relatedness based on published phylogenies [38, 39]. We implemented this method directly in the extraction sheet using Excel formulas. Additional file 1: Table S1 illustrates the online tools used in this method and Additional file 1: Table S2 and Additional file 5 detail how individual genotypes were related based on this method.

#### Data quality checks

We performed several data quality checks using code written in R software (version 3.3.3) to check for duplicate extractions and for discrepancies between the screening and extraction sheets. We checked that all extracted studies were included in the screening sheet, and vice versa, using PubMed ID or a unique study identifier. We checked for duplicate extractions by looking for any studies that (1) had duplicate PubMed ID or unique study identifiers, (2) had the same country and start year, or (3) had the same country and sample size. Each potentially duplicated or missed study was checked manually, and the decision to include or exclude was recorded.

#### Proportion of estimated TB cases represented in each country

We determined the proportion of estimated TB cases that were represented in each country for which we had data using estimates from the Global Burden of Disease Study 2016 [40]. We downloaded estimates of the total number of TB cases prevalent in each country and year across all ages and sexes from https://vizhub.healthdata.org/gbd-compare/ (accessed on July 29, 2018). We matched these country-year estimates to each study based on the country the study was conducted in and the year that corresponded to the mid-point of its sampling period. We divided the sample size of each study by the estimated prevalent TB cases in the corresponding country and year to get the proportion represented in each study. We then summed the proportions represented in all studies within each country to get final estimates of the proportion of TB cases represented per country.

#### Map of the global distribution of genotypes

We determined the proportion of each phylogenetic lineage present in each country for which we had data. If multiple studies were available in a country, we summed the strain counts across all studies and years to get the final proportions and sample sizes.

#### Meta-analysis of genetic clustering association with genotypes

We performed a random effects (RE) meta-analysis of the relative risk (RR) of genetic clustering associated with genotypes using the Reliability Method (RELM) method in the R software package “metafor” (version 3.3.3) [41]. We excluded studies that identified fewer than two isolates of the lineages under analysis. We examined inconsistency across studies using the *I*^2^ test that measures the percentage of total variation across studies due to heterogeneity [42]. We performed a subgroup analysis of genetic clustering within the regions West Asia, East Asia, Europe and the Americas, and Africa.

## Results

### Study selection

**Fig. 1:**
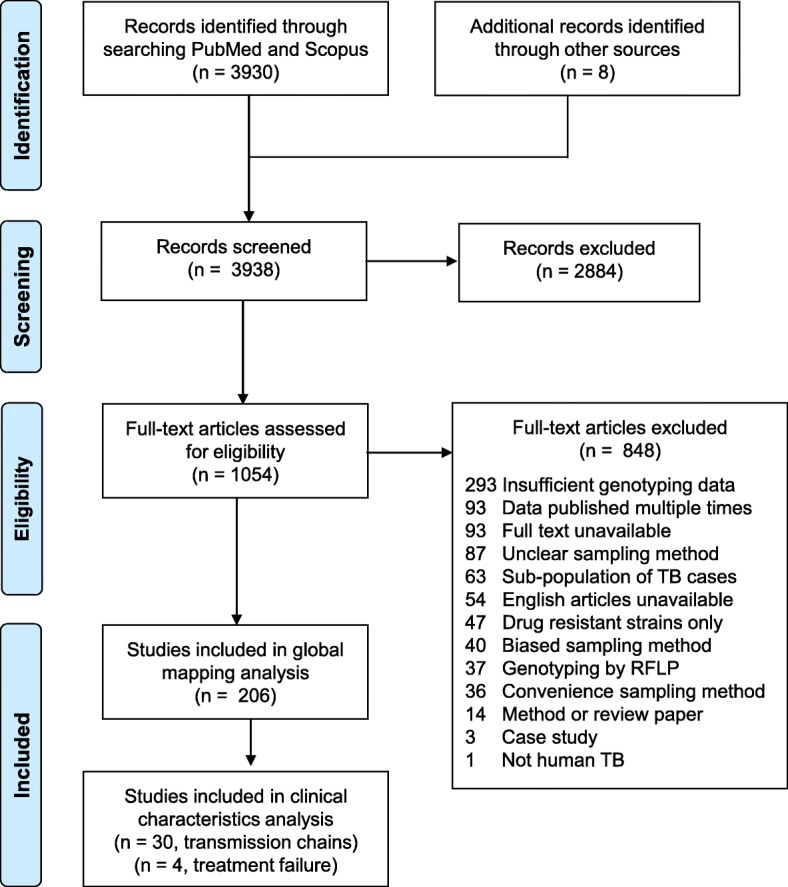
PRISMA flow diagram. Diagram illustrating the literature selection process, including identification, screening, eligibility, and total studies included in the global analysis and clinical characteristic analysis. Reasons for exclusion of full texts are detailed. Individual-level details of each study reviewed are found in Additional file 2

We identified 206 studies for inclusion in the study, representing over 200,000 bacterial isolates collected over 27 years in 85 countries. Of these studies, 30 also reported transmission chains and 4 reported treatment failure associated with genotypes. Figure 1 shows the PRISMA flow diagram detailing the study selection process. Additional file 1: Figure S1 shows a map of the numbers of studies per year that were included from each country.

### Study variation

**Fig. 2:**
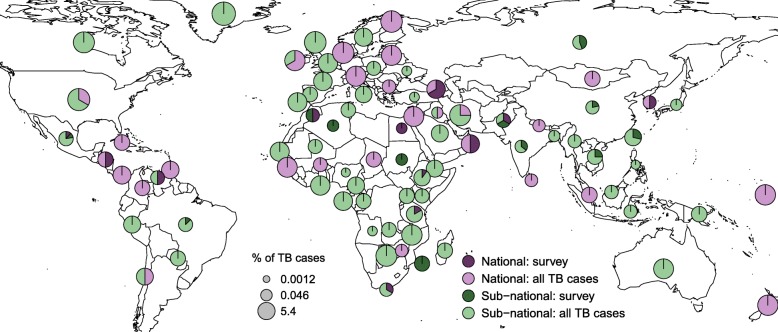
Variation in sampling methods of studies included in the systematic review. Variation in study design for the 206 studies that met the inclusion criteria for this systematic review. The proportion of studies in each country that collected a nationally representative sample versus a sample representative of a smaller geographic location are shown in purple and green, respectively. Light purple and green indicate the proportion of studies in each country that collected all reported or all new TB cases in a given location and time period. For the majority of studies, “all TB cases” represents culture-positive cases only; for studies that use GeneXpert remnants for DNA collection, this represents microscopy-positive cases. Dark purple and green indicate the proportion of studies in a given country that used a random or cluster-based survey sampling method to select a subset of cases. TB cases in each country were estimated by the Global Burden of Disease Study 2016 [40]. We calculated percent of all TB cases in each country using the total number of genotyped cases as the numerator and total estimated prevalent TB cases as the denominator. The radius of each pie is proportional to percent of total estimated TB cases that are represented across all studies in each country. Examples of percent of total estimated TB cases that correspond to pie sizes are shown in the legend in gray. The example pies show the minimum, mid-point, and maximum percent of estimated TB cases represented in this review

The 206 studies included 42 nationally representative samples and 164 samples representative of smaller geographic units. These included 34 studies that used a cluster or random sampling, 170 that collected samples from all reported or new TB cases in a given geographic location and time period, and 2 studies that used different sampling methods for different time periods. We illustrated how these study designs varied globally in Fig. 2. Sub-Saharan Africa was dominated by subnationally representative studies, while Caribbean Latin America was dominated by nationally representative studies (Fig. 2). In addition, we calculated the proportion of estimated prevalent TB cases that were represented in each country. Proportions ranged from 0.0012% in Nigeria to 5.4% in Greenland (Fig. 2). In general, the proportions were lower in countries where TB burden was highest (Fig. 2). The meta-data linked with each individual study is available in its raw format in Additional file 3.

### Geographic variation in MTBC genotypes

**Fig. 3:**
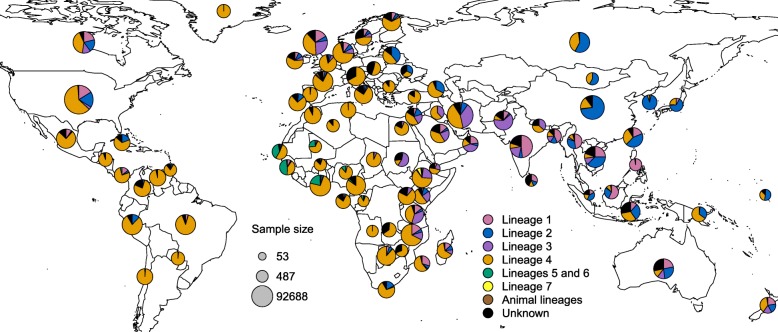
The global distribution and genetic diversity and of MTBC phylogenetic lineages. MTBC global genotype distribution by country across all years based on a systematic review of TB molecular epidemiology studies employing one of four genotyping methods: (1) spoligotyping, (2) MLVA typing, (3) PCR typing for large sequence polymorphisms, and (4) whole-genome sequencing. All genotyping methods are converted to a common classification system based on phylogenetic lineages (Additional file 1: Tables S1 and S2), and pie charts show the proportion of lineages present in each country where data was available and studies met our inclusion criteria. Indo-Oceanic lineage 1 is shown in pink, lineage 2 is shown in blue, East African-Indian lineage 3 is shown in purple, Euro-American lineage 4 is shown in orange, West African lineages 5 and 6 are shown in green, and Ethiopian lineage 7 is shown in yellow. “Unknown” represents strain types that were not identified by the authors either due to low frequency or unknown genetic patterns. Studies that report prevalence of only one lineage and grouped all other genotypes as “other” are excluded from the map. If multiple studies were available in a country, strain counts were summed across all studies to get final proportions and sample sizes. The radius of each pie is proportional to the number of isolates collected in each country. Examples of sample sizes that correspond to pie sizes are shown in the legend in gray. The example pies shown represent the minimum, mid-point, and maximum samples sizes

**Fig. 4:**
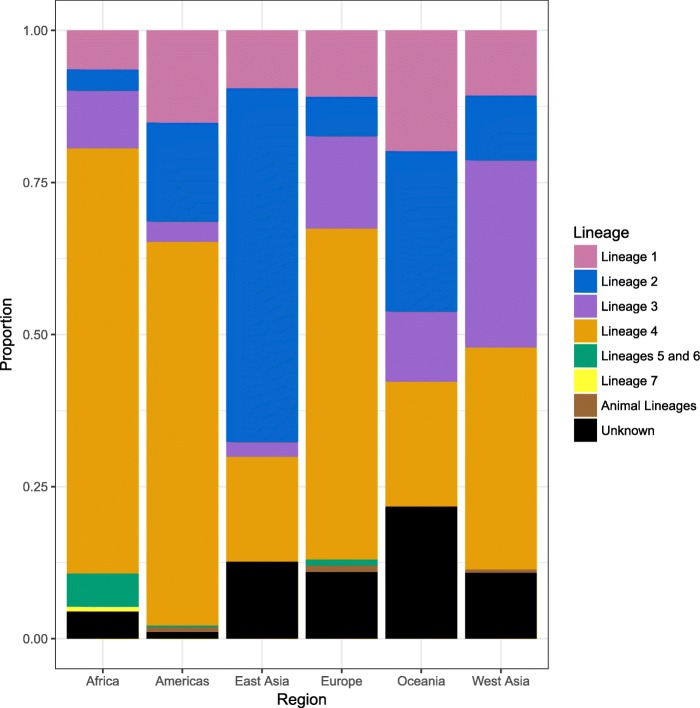
Distribution of MTBC phylogenetic lineages by region. MTBC global genotype distribution by region corresponding to the data presented in Fig. 3. Lineage proportions broken down by countries within each region are shown in Additional file 1: Table S3 and Figure S2

We mapped the distribution of MTBC genotypes identified in our systematic review across all years and for all locations in each country for which we had data. A striking feature of the map was the widespread global distribution of Euro-American lineage 4 (Figs. 3 and 4). Lineage 4 was identified in every country where genotyping data was available for inclusion, and it was the majority lineage in 52 of the 85 countries (Additional file 1: Table S3). Our map also showed the fairly widespread distribution of East Asian lineage 2 (Figs. 3 and 4), which was identified in 67 of the 85 countries and was the majority lineage in 6 countries (Additional file 1: Table S3). In contrast, West African lineages 5 and 6 were identified in only 30 countries and were the majority lineages in zero countries (Additional file 1: Table S2). In addition, Indo-Oceanic lineage 1 and East African-Indian lineage 3 were identified in 64 and 59 countries, respectively, and each was the majority lineage in 2 countries (Additional file 1: Table S3).

The map also illustrated various regions of distinct mycobacterial distribution that may be independent of geopolitical country boundaries (Fig. 3 and Additional file 1: Figure S2). For example, Eastern Africa from Sudan to Mozambique was distinct from the rest of Africa in that it had a higher prevalence of lineages 1 and 3. Western Africa was distinct in that it had the highest prevalence of lineages 5 and 6, while Southern Africa had the highest prevalence of lineage 2 strains and Central Africa had the highest prevalence of lineage 4 strains. In addition, the Indian subcontinent and Australia had a similar genotype distribution, which was distinct from Russia and Eastern Asia. The UK was distinct from the rest of Europe in that it had a greater prevalence of lineages 1 and 3. Finally, Central America and northern South America had distinct genotype distributions from central and southern South America.

### Temporal variation in MTBC genotypes

**Fig. 5:**
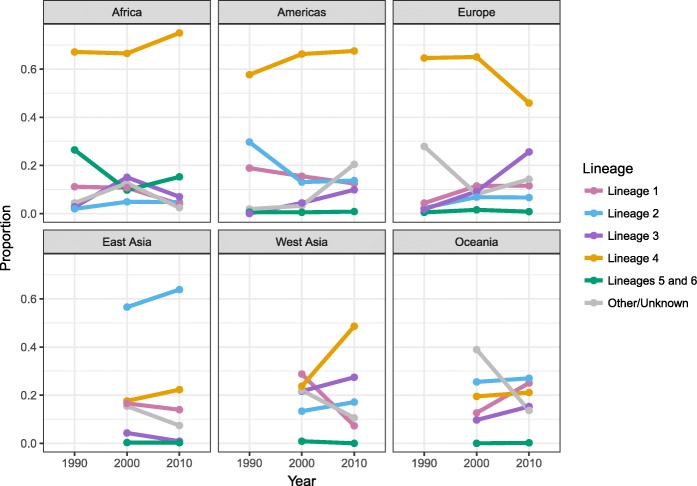
Distribution of MTBC lineages over time by region. MTBC genotype distribution by region over time corresponding to results presented in Additional file 1: Figure S3. The year 1990 represents all studies from 1990 to 1999, the year 2000 represents all studies from 2000 to 2009, and the year 2010 represents all studies from 2010 to 2017. Indo-Oceanic lineage 1 is shown in pink, East Asian lineage 2 is shown in blue, East African-Indian lineage 3 is shown in purple, Euro-American lineage 4 is shown in orange, and West African lineages 5 and 6 are shown in green. Other/unknown strains are shown in gray and represent animal lineages, lineage 7, and strain types that were not identified by authors either due to low frequency or unknown genetic patterns. Strain counts and sample sizes were summed across all studies within the given regions and time periods to get proportions. There was no data from East Asia, West Asia, and Oceania in the 1990s, and therefore, these years are left blank

The results described above represent MTBC genotype distributions aggregated across all years from 1990 to 2017. In order to investigate the changes over time in genotype distribution, and to illustrate the time periods that more accurately represented the data in each country, we created maps of genotype distribution for three distinct time periods (Additional file 1: Figure S3). To synthesize these data, we plotted the total prevalence of each lineage in each time period by region (Fig. 5). Figure 5 should be used as a guide and interpreted with some caution as it represents data aggregated across diverse geographic locations. The plots showed that lineage 3 strains have increased in prevalence over time in the UK (Additional file 1: Figure S3A-C) and Europe (Fig. 5). In addition, the plots showed a decline in the prevalence of lineage 1 in West and Central Asia (Fig. 5 and Additional file 1: Figure S3B-C).

### Clinical variation in MTBC genotypes

#### Transmission chains as measured by genetic clustering

We performed a random-effects meta-analysis [41] of the 30 studies that reported transmission chains or genetic clusters associated with MTBC genotypes. We defined genetic clusters as two or more identical genotype patterns identified in the same study location and time period. We used lineage 4 as the reference group because lineage 4 strains were identified in each study included in the meta-analysis. The characteristics of each study included in the meta-analyses are shown in Additional file 1: Table S4. We analyzed transmission chain relative risk (RR) across all studies, as well as within subgroups of Africa, East Asia, West Asia, and Europe and the Americas.

The results of the meta-analyses are summarized in Table 1, and detailed forest plots are shown in Additional file 1: Figure S4. Lineage 1 strains overall were not associated with transmission chains (RR [95% CI] = 1.07 [0.83, 1.37]) (Table 1) but were associated with increased risk within East Asia (RR [95% CI] = 2.54 [1.02, 6.28]) (Additional file 1: Figure S4A). Lineage 2 Beijing strains were associated with increased risk of transmission chains overall (RR [95% CI] = 1.24 [1.07, 1.45]) (Table 1), and the risk was higher within East Asia (RR [95% CI] = 1.90 [1.14, 3.17]) (Additional file 1: Figure S4B). Lineage 3 strains were associated with reduced risk of transmission chains in Europe and the Americas (RR [95% CI] = 0.67 [0.50, 0.91]) (Additional file 1: Figure S4C). Lineages 5 and 6 strains were associated with reduced risk of transmission chains overall (RR [95% CI] = 0.61 [0.43, 0.86]) (Table 1, Additional file 1: Figure S4D), as were animal lineage strains (RR [95% CI] = 0.79 [0.64, 0.96]) (Table 1, Additional file 1: Figure S4E). Unknown strains, which comprise orphans, undefined, and uncommon genotypes, were associated with reduced risk of transmission chains overall (RR [95% CI] = 0.56 [0.40, 0.79]) (Table 1, Additional file 1: Figure S4F).

**Table 1:** Summary of random effects (RE) meta-analyses of relative risk (RR) of transmission chains associated with MTBC lineages

Lineage number	Lineage name	RR	*p* value (RR)	CI, lower	CI, upper	*Q*	*p* value (*Q*)	*I*^2^ (%)	Number of studies
4	Euro-American	1.00							
1	Indo-Oceanic	1.07	0.61	0.83	1.37	228.3	0.00	95.4	17
2	East Asian	1.24	0.01	1.07	1.45	220.6	0.00	97.8	20
3	East African-Indian	0.98	0.84	0.81	1.18	130.6	0.00	84.9	17
5, 6	West African	0.61	0.01	0.43	0.86	39.4	0.00	95.0	8
–	Animal	0.79	0.02	0.64	0.96	7.43	0.28	18.0	7
–	Unknown	0.56	0.00	0.40	0.79	70.5	0.00	92.0	12

RE meta-analysis of the RR of transmission chains associated with each MTBC lineage compared with MTBC lineage 4. Transmission chains in this analysis are defined as identification of two or more MTBC isolates with identical genetic patterns in the same study location and time period. “Cluster” indicates part of a transmission chain, and “unique” indicates not part of a transmission chain. Lineage 7 strains are grouped with “unknown” strains because there was insufficient data on these strains for meta-analysis. We performed the analysis across all studies that we identified in the systematic review, as well as within the regions West Asia, East Asia, Europe and the Americas, and Africa. RE meta-analysis was performed using the RELM method in R software package “metafor” (version 3.3.3) [41]. Forest plots for each analysis are shown in Additional file 1: Figure S4A-F.

These results should be interpreted with some caution as *I*^2^ analysis showed significant heterogeneity across all studies (Table 1), as well as within most subgroups (Additional file 1: Figure S3), with a few exceptions. There was low heterogeneity between studies in the animal strains analysis (Table 1, *I*^2^ = 18%), as well as in the analysis of lineages 5 and 6 strains within Europe and the Americas (Additional file 1: Figure S3D, *I*^2^ = 0.0%).

#### Treatment failure

Several studies identified in the systematic review showed that lineage 2 Beijing family strains were associated with treatment failure. Beijing strains were associated with treatment failure in Indonesia compared with all other genotypes after adjusting for drug resistance, non-adherence, age, diabetes mellitus, and severity of radiological lesions (relative risk [95% CI] = 1.94 [1.26, 3.0]) (Table 2) [43]. Beijing strains were also associated with treatment failure after adjusting for multi-drug resistance in India (odds ratio [95% CI] = 3.29 [1.29, 8.14]) (Table 2) [44]. However, Beijing strains were not associated with treatment failure after adjusting for multi-drug resistance in Vietnam (odds ratio [95% CI] = 0.7 [0.3, 2.0]) (Table 2) [45] and were not associated with treatment failure of drug-susceptible TB in South Africa (Table 2) [46]. Confounders that were not adjusted for in all these studies, such as HIV co-infection, diabetes mellitus, body mass index (BMI), cavitary TB, and quality of health care, may contribute to the variation in results (Table 2).

**Table 2:** Summary of treatment failure studies

Source	Location	Years	No. of patients	Confounders adjusted for	Findings	RR/OR [95% CI]
Parwati et al. [43]	Indonesia	2000–2005	818	Drug resistance, non-adherence, age, diabetes mellitus, and severity of radiological lesions	Lineage 2 Beijing family were associated with treatment failure.	RR 1.94 [1.26, 3.0]
Chatterjee et al. [44]	India	2004–2007	646	Multi-drug resistance	Lineage 2 Beijing family were associated with treatment failure.	OR 3.29 [1.29, 8.14]
Buu et al. [45]	Vietnam	2003–2007	1106	Multi-drug resistance	Lineage 2 Beijing family were not associated with treatment failure.	OR 0.7 [0.3, 2.0]
van der Spuy et al. [46]	South Africa	1993–2004	1737	None (analysis included only drug-susceptible strains)	Lineage 2 Beijing family were not associated with treatment failure.	Not reported (*p* > 0.05)

Summary of study design and findings for each study reported genotype associations with treatment failure. RR indicates relative risk, OR indicates odds ratio, and 95% CI indicates 95% confidence interval. The latter measures were taken directly from the studies and were not reanalyzed.

## Discussion

To our knowledge, this study represents the most comprehensive dataset on MTBC lineages that has been created by systematically assembled genotyping data from studies that used representative sampling techniques. The data show geographic variation in MTBC genotypes, which is consistent with previously published studies that used convenience samples and much smaller datasets. We find some evidence for clinical variation between genotypes, though, we also show significant variation between studies, which highlights the need for additional data.

### Global variation in bacterial strains that cause TB disease

The results presented in this study are consistent with previously published maps that showed that MTBC strains that evolved more recently in human history—lineage 2, lineage 3, and lineage 4 strains—tend to be more widely distributed around the world [22, 35, 47, 48]. We also showed that lineage 1, lineage 2, and lineage 3 are more prevalent in Europe and in North and South America than shown in previously published maps [35, 47, 48]. Moreover, we show that lineage 3 strains may be increasing in prevalence in Europe, while lineage 1 strains may be decreasing in prevalence in West Asia. These patterns in genotype distribution likely reflect both historical and recent movement of strains with people from East Asia and the Indian subcontinent to Europe and the American continent. The dominance of lineage 4 globally, and in particular in South American countries, also supports the hypothesis that European colonialists aided in the dispersion of this lineage in the mid-sixteenth to nineteenth centuries [32, 48, 49]. If the first inhabitants of the American continent brought early forms of lineage 2 strains with them when they migrated from north-eastern Asia, these strains may have been eliminated with the arrival of strains from European colonialists.

Human migration is likely not the only determinant of MTBC genotype distribution. Lineages 5 and 6 are prevalent only in West Africa [35, 47, 48]. The reasons for this geographic restriction are largely unknown but may have to do with clinical characteristics of the patients infected with these strains. Patients infected with lineage 6 are more likely than patients infected with other strains to be older, HIV-infected, and severely malnourished [50]. In addition, we showed that lineages 5 and 6 strains may be less likely to cause transmission chains than lineage 4 strains and that these findings were more consistent in Europe and the Americas than in Africa, which may reflect biological differences and/or social mixing which prevents these strains from spreading through non-West African populations. We also found that lineage 3 strains were associated with reduced risk of transmission chains in Europe and the Americas, which is consistent with the findings from a household contact study in Montreal [51]. In contrast, we found that Beijing family strains may be more likely to cause transmission chains, which could reflect the ability of Beijing strains to spread quickly through human populations [46, 52, 53]. These findings are not consistent with previous work that showed no differences between lineages in transmission from household contacts [46, 54, 55]. Thus, further studies would be required to confirm our findings.

Several studies included in our analysis showed that treatment failure was associated with lineage 2 Beijing family strains [43, 44]. Beijing family strains are also associated with drug resistance [56], which has been reviewed previously [12, 22, 23]. Additionally, lineage 1 strains have been associated with more rapid response to treatment in drug-susceptible TB cases in the USA [57]. Thus, there is evidence for a relationship between bacterial genotype and treatment outcome, at least in certain populations or contexts. Future studies that carefully control for potential confounders that may impact treatment failure are required to confirm these findings. This type of information could be particularly important to clinicians if it could inform the development of novel diagnostic tools that test for bacterial genotypes associated with poor response to treatment and development of drug resistance.

### Variation between studies and implications for variation in MTBC genotypes

There was variation in the sampling methods and representativeness of the studies included in this systematic review. The majority of studies were representative of much smaller geographic locations than the national level, and despite the large number of bacterial isolates included in this study, they represented only a small fraction of the total estimated TB cases. While the goal of this study was to summarize the MTBC genotyping data available, not to make nationally representative estimates, it is important to note that this variation was not distributed evenly throughout the world. There was less information available about MTBC genotype distribution in South America and Sub-Saharan Africa than in other regions, and the data in Central and Eastern Asia represented a smaller proportion of all estimated TB cases than elsewhere. Thus, the genetic diversity shown in the map in Fig. 3 for these regions is likely less representative of the underlying populations.

Another source of variation that may impact representativeness is whether studies were biased towards including either rural or urban populations. There is likely greater MTBC genetic diversity in patients from urban populations than patients from rural areas since urban areas experience higher rates of travel and migration. Most studies included in this analysis did not report the urban/rural composition of their sample, and the bias towards one or the other would likely vary depending on study location. For example, the majority of the studies included in our systematic review used samples collected from public hospitals or reference laboratories. Therefore, in countries such as India, where people in urban areas may be more likely to seek care from private health clinics [58], the urban population may be underrepresented and we may have underestimated genetic diversity. On the other hand, in countries such as Uganda, where the rural population has limited access to public health facilities [59], the rural population may be underrepresented and we may have overestimated genetic diversity. This highlights the importance of data from prevalence surveys that use active surveillance techniques to reach a broader subset of the population.

We also identified a significant amount of heterogeneity between studies in the meta-analysis of genetic clustering associated with genotypes. One source of this heterogeneity is likely methodological differences between the studies, such as genotyping method, sampling method, and study duration, which have been shown to impact genetic clustering [27, 28]. For example, duration of sampling ranged from 2 months to 9 years, and genotyping methods ranged from the use of either spoligotyping or MLVA typing to the use of both methods (Additional file 1: Table S4). Studies that used shorter sampling durations may have missed transmission chains and underestimated clustering, while studies that used spoligotyping only may have overestimated clustering [60]. An additional source of heterogeneity may be confounders that impact genetic clustering and transmission, such as social mixing, immigration, age structure, comorbidities, and underlying TB incidence [27, 28]. These confounders likely also varied between these studies but were often not reported. For example, only 14 of the studies reported HIV prevalence (range 0 to 91%), only 6 reported proportion of immigrants (range 0 to 78%), and only 14 reported mean age of patients (range 25 to 50) included in the sample (Additional file 1: Table S4). If social mixing was high in each of the studies, this could have led us to overestimate the impact of genotype on transmission chains, while if migration was high, this could have led us to underestimate the presence of transmission chains.

### Study limitations

A limitation of this study is that we grouped strains into seven lineages, which masks within-lineage variation. Distinct sub-lineages of the Beijing family are associated with differences in transmissibility in human populations [61, 62], and lineage 4 contains both geographically widespread and restricted sub-lineages [49]. However, we propose that this was the best method as it allowed us to (1) include a broad range of studies, including those that did not report sub-lineages, and (2) synthesize studies that used WGS- or PCR-based typing together with studies that used methods more common in resource-limited settings, such as spoligotyping and MLVA typing.

Another limitation is that we did not include data from WGS databases. A challenge of incorporating WGS data is identifying study meta-data, such as sampling methods and demographic characteristics of patients, linked with genomes. In addition, many of the WGS data available are poised for phylogeographic studies and for examining the presence of specific mutations [32, 49, 56], but are less representative of the populations they are isolated from. These data are often from outbreaks or studies of specific sub-populations, which we excluded in this analysis. As WGS data linked with meta-data become more available (through prevalence surveys [63] and endeavors such as ReSeqTB) including this data would be an important extension of our study. Our study supports these future studies by illustrating the importance of using genome sequences to determine phylogenetic lineages or sub-lineages. The dataset we have created could be used to fill geographic gaps in future WGS-based maps, particularly in regions where WGS technology is unavailable, and to verify results from convenience-based samples.

## Conclusions

The evidence gathered in this systematic review support a role for bacterial genetic diversity in understanding global variation in TB disease. However, there are aspects of the studies that restrict our ability to confidently attribute clinical characteristics to genotypes. In order to address these conditions in the future, there will need to be a shift in the design of MTBC strain diversity studies such that data is collected in a way that is clinically and epidemiologically informative, wherever possible. We encourage future studies to carefully consider potential confounding variables in study design and analysis and to make all genotypes and study meta-data publicly available upon publication. We also encourage the analysis of less-studied strains from lineages 1 and 3 in order to increase comparability with the relative abundance of data on lineage 2 and lineage 4 strains. The evidence presented in this study demonstrate these types of data could potentially be used to create tools to inform the clinical diagnosis and treatment of TB and improve our understanding of the epidemiology of this disease.

## Additional files

Additional file 1: Supplementary appendix. Document containing complete description of literature search strings and dates searched, as well as Tables S1-S4 and Figure S1-S4. (PDF 1440 kb)

Additional file 2: Literature screening sheet. Literature screening sheet including citation information for all literature included in the study. (XLSX 3109 kb)

Additional file 3: Raw genotype distribution data. Raw genotype distribution data extracted in the systematic review. (CSV 1975 kb)

Additional file 4: Raw genetic clustering data. Raw genetic clustering data extracted in the systematic review. (CSV 15 kb)

Additional file 5: Genotype classification system. Sheets containing MTBC genotype conversions for all genotyping methods included in this study. (XLSX 146 kb)

## Abbreviations

CI: Confidence interval
LSP: Large sequence polymorphism
MIRU: Mycobacterial interspersed repetitive units
MLVA: Multi-locus variable number of tandem repeats analysis
MTBC: *Mycobacterium tuberculosis* complex
OR: Odds ratio
PCR: Polymerase chain reaction
PRISMA: Preferred Reporting Items for Systematic Reviews and Meta-Analyses
RE: Random effects
RFLP: Restriction fragment length polymorphism
RR: Relative risk
TB: Tuberculosis
VNTR: Variable number of tandem repeats
WGS: Whole-genome sequencing
