# Supplementary material for: Global variation in bacterial strains that cause tuberculosis disease: a systematic review and meta-analysis
Source: BMC Med. 2018 Oct 30;16:196. doi: 10.1186/s12916-018-1180-x (PMC6206891; doi:10.1186/s12916-018-1180-x)

Table of Contents

Systematic review search strings 2
Supplementary tables 3
Table S1. Tools to convert MTBC genotypes to a common classification scheme 3
Table S2. Common classification scheme for MTBC strains 4
Table S3. Genetic diversity of MTBC strains by country 5
Table S4. Summary of studies included in random effects (RE) meta-analysis of genetic clustering 9
Supplementary figures 11
Figure S1. Human TB molecular epidemiology studies 1990-2017 11
Figure S2. Genetic diversity and global distribution of MTBC phylogenetic lineages. 12
Figure S3. Global distribution and genetic diversity and of MTBC phylogenetic lineages over time. 13
Figure S3A. MTBC global genotype distribution by country 1990-1999. 13
Figure S3B. MTBC global genotype distribution by country 2000-2004. 14
Figure S3C. MTBC global genotype distribution by country 2005-2009. 14
Figure S3D. MTBC global genotype distribution by country 2010-2017. 15
Figure S4. Random effects (RE) meta-analyses of the relative risk (RR) of transmission chains associated with MTBC lineages 16
Figure S4A. RR of transmission chains for lineage 1 strains. 17
Figure S4B. RR of transmission chains for lineage 2 Beijing strains. 18
Figure S4C. RR of transmission chains for lineage 3 strains. 19
Figure S4D. RR of transmission chains for lineage 5 and 6 strains. 20
Figure S4E. RR of transmission chains for animal strains. 21
Figure S4F. RR of transmission chains for unknown strains. 22

Systematic review search strings

We searched PubMed and Scopus using the search strings detailed below. Articles identified from these searches were reviewed for eligibility first by screening titles and abstracts and then by reviewing full texts. Articles identified in these searches were supplemented by articles we were directed to by collaborators, as detailed in the Methods section of the main text.

Database	Date	Search string
PubMed	06/08/17	((TB[TIAB] OR tuberculosis[TIAB] OR "Mycobacterium tuberculosis"[TIAB] OR "M tuberculosis"[TIAB] OR "Mycobacterium africanum"[TIAB] OR "M africanum"[TIAB] OR "Mycobacterium tuberculosis"[MeSH] OR "Tuberculosis"[MeSH]) AND (spoligotyp*[TIAB] OR spoligo[TIAB] OR “whole genome sequencing”[TIAB] OR WGS[TIAB] OR LSP*[TIAB] OR “large sequence polymorphism”[TIAB] OR “large sequence polymorphisms”[TIAB] OR MIRU[TIAB] OR VNTR[TIAB] OR “mycobacterial interspersed repetitive units”[TIAB] OR “mycobacterial interspersed repetitive unit"[TIAB] OR “variable number of tandem repeats”[TIAB] OR “variable number tandem repeats”[TIAB] OR "variable number tandem repeat”[TIAB] OR MLVA[TIAB] OR “restriction fragment length polymorphism”[TIAB] OR RFLP[TIAB] OR genotyp*[TI])) NOT (Animals[MeSH] NOT Humans[MeSH])
Scopus	09/01/17	((TITLE-ABS-KEY(TB) OR TITLE-ABS-KEY(tuberculosis) OR TITLE-ABS-KEY("Mycobacterium tuberculosis") OR TITLE-ABS-KEY ("M tuberculosis") OR TITLE-ABS-KEY **(**"Mycobacterium africanum") OR TITLE-ABS-KEY("M africanum")) AND (TITLE-ABS-KEY(spoligotyp*) OR TITLE-ABS-KEY(spoligo) OR TITLE-ABS-KEY(“whole genome sequencing”) OR TITLE-ABS-KEY(WGS) OR TITLE-ABS-KEY(LSP*) OR TITLE-ABS-KEY(“large sequence polymorphism”) OR TITLE-ABS-KEY(“large sequence polymorphisms”) OR TITLE-ABS-KEY(MIRU) OR TITLE-ABS-KEY(VNTR) OR TITLE-ABS-KEY(“mycobacterial interspersed repetitive units”) OR TITLE-ABS-KEY(“mycobacterial interspersed repetitive unit") OR TITLE-ABS-KEY(“variable number of tandem repeats”) OR TITLE-ABS-KEY(“variable number tandem repeats”) OR TITLE-ABS-KEY("variable number tandem repeat”) OR TITLE-ABS-KEY(mlva) OR TITLE-ABS-KEY(“restriction fragment length polymorphism”) OR TITLE-ABS-KEY(RFLP) OR TITLE(genotyp*))) AND NOT (TITLE-ABS-KEY(cattle) OR TITLE-ABS-KEY(livestock) OR TITLE-ABS-KEY(wildlife) OR SUBJAREA(VETE) OR DBCOLL(medl))
PubMed	11/13/17	((TB[TIAB] OR tuberculosis[TIAB] OR "Mycobacterium tuberculosis"[TIAB] OR "M tuberculosis"[TIAB] OR "Mycobacterium africanum"[TIAB] OR "M africanum"[TIAB] OR "Mycobacterium tuberculosis"[MeSH] OR "Tuberculosis"[MeSH]) AND (spoligotyp*[TIAB] OR spoligo[TIAB] OR “whole genome sequencing”[TIAB] OR WGS[TIAB] OR LSP*[TIAB] OR “large sequence polymorphism”[TIAB] OR “large sequence polymorphisms”[TIAB] OR MIRU[TIAB] OR VNTR[TIAB] OR “mycobacterial interspersed repetitive units”[TIAB] OR “mycobacterial interspersed repetitive unit"[TIAB] OR “variable number of tandem repeats”[TIAB] OR “variable number tandem repeats”[TIAB] OR "variable number tandem repeat”[TIAB] OR MLVA[TIAB] OR “restriction fragment length polymorphism”[TIAB] OR RFLP[TIAB] OR genotyp*[TI])) NOT (Animals[MeSH] NOT Humans[MeSH])

Supplementary tables

Table S1. Tools to convert MTBC genotypes to a common classification scheme. Common genotyping methods and tools used to convert them to a common classification system based on phylogenetic lineages in this review.

Convert from	Convert to	Tool	Link	Input	Output
Spoligotype	Phylogenetic lineage	TB-Lineage	http://tbinsight.cs.rpi.edu/run_tb_lineage.html	- Octal or binary spoligotypes in tab-delimited file<br>- Choice of Rules or CBN method	- Phylogenetic lineage name<br>- Probability (if using CBN)
Spoligotype clade	Phylogenetic lineage	Supplementary file 5	-	Filter by spoligotype clade in sheet	Phylogenetic lineage name
MLVA type	Spoligotype<br>MLVA lineage<br>LSP	MIRU-VNTR *plus*	http://www.miru-vntrplus.org/MIRU/index.faces	12, 14, or 24-loci MIRU-VNTR types in Excel or CSV file	- Species<br>- MLVA MtbC15-9<br>- SpolDB4 Type<br>- Spoligotype<br>- LSP and SNP
MLVA lineage	Phylogenetic lineage	Supplementary file 5	-	Filter by MLVA lineage in sheet	Phylogenetic lineage name
LSP	Phylogenetic lineage	Gagneaux *et al.* Lancet Infect Dis 2007; 7: 328–37	http://dx.doi.org/10.1016/S1473-3099(07)70108-1	Identify region of difference (RD) number in Figure 1	Phylogenetic lineage name
LSP	Phylogenetic lineage	Supplementary file 5	-	Filter by LSP number in sheet	Phylogenetic lineage name

Table S2. Common classification scheme for MTBC strains. Common genotypes and how they were converted to a common classification system based on phylogenetic lineages in this review. Detailed conversion of all genotypes identified in this study are available in Additional file 5.

Phylogenetic lineage number	Phylogenetic lineage name	LSP number	MLVA lineages	Spoligotype clades
1	Indo-Oceanic	239	EAI, Indo-Oceanic	EAI, EAI1, EAI1-SOM, EAI2, EAI2-Manila, EAI2-Nonthaburi, EAI2-NTB, EAI3, EAI3-IND, EAI4, EAI4-VNM, EAI5, EAI6-BGD1, EAI7-BGD2, EAI8-MDG, Manu_ancestor, Manu1
2	East Asian	105, 142, 150, 181, 207	Beijing, East-Asian, ST11/26, ST25/19, ST3, STK	Beijing, Beijing-like
3	East African-Indian	750	CAS, Delhi/CAS	CAS, CAS1, CAS1-DAR, CAS1-Delhi, CAS1-Kili, CAS2, CAS-KILI, H2, KILI, U(CAS_ANCESTORO)
4	Euro-American	115, 122, 174, 182, 183, 193, 219, 724, 726, 761	Cameroon, Congo, Ethiopia_2, Ethiopia_H37rvlike, Euro-American, Ghana, H37Rv, H37Rv-like, Haarlem, LAM, NEW-1, NW-ETH2, NW-ETH4, S, T, T or undefined, TUR, Uganda I, Uganda II, URAL, X	Ambiguous:T2 T5, Ambiguous:T3 T2, Ambiguous:T4 T2, Cameroon, EAI1-SOM, Euro-American, F33, Family33, Family36, Gambian family, Ghana, H, H1, H1-S, H3, H3-T3, H37, H37Rv, H37Rv-like, H3-Ural-1, H4, H4-Ural-2, Haarlem, Haarlem1, Haarlem2, Haarlem3, LAM, LAM1, LAM10, LAM10-CAM, LAM11-ZWE, LAM12-Madrid1, LAM2, LAM3, LAM4, LAM5, LAM6, LAM7, LAM7-TUR, LAM8, LAM9, Manu, Manu2, Manu3, S, T, T1, T1 RUS1, T1-RUS2, T1T2, T2, T2-T3, T2-Uganda, T3, T3-ETH, T3-OSA, T4, T4-CEU1, T5, T5-Madrid2, T5-RUS1, T-H37Rv-3, TS, T-Tuscany, TUR, Turkey, U/H3, Uganda I, Uganda II, Ural, X, X1, X1-LAM9, X2, X3, ZERO
5, 6	West African	702, 711	Africanum, M. africanum, West African, West African 1, West African 2	AFRI, AFRI_1, AFRI_2, AFRI_3, West Africa I, West Africa II
7	Ethiopian	-	Ethiopia_1, Ethiopia_3, Lineage 7, NW-ETH1, NW-ETH3	-
-	Animal	7, 8, 10 (M. bovis)	Bovis, Caprae, Llama, M. bovis, M. bovis BCG, M. caprae, M. microti, M. origys, M. pinnipedi, Seal, Vole	BOV, BOV_1, BOV_2, BOV_3, BOV_4-Caprae, BOVIS, Microti, PINI, PINI1, PINI2, PINI2
-	Other	-	Undefined, Unknown, Other	CZ-ST 1, CZ-ST 2, CZ-ST 3, CZ-ST 4, CZ-ST 5, CZ-ST 6, Mixed, NA, ND, New, Other, U, Undefined, Unknown, Vietnam type

Table S3. Genetic diversity of MTBC strains by country. Total counts of isolates and proportions (shown in parentheses) of MTBC phylogenetic lineages collected in each country where data was available for inclusion in the systematic review. Raw data can be found in Additional file 3.

Location	Lineage 1	Lineage 2	Lineage 3	Lineage 4	Lineage 7	Animal	Lineage 5 & 6	Other	Total
Africa	*1489 (0.064)*	*812 (0.035)*	*2232 (0.095)*	*16350 (0.699)*	*162 (0.007)*	*40 (0.002)*	*1279 (0.055)*	*1030 (0.044)*	*23394*
**Algeria**	0	0	0	114 (0.884)	0	0	0	15 (0.116)	129
**Angola**	0	0	0	88 (1)	0	0	0	0	88
**Botswana**	79 (0.049)	98 (0.061)	10 (0.006)	1428 (0.884)	0	1 (0.001)	0	0	1616
**Burkina Faso**	4 (0.033)	0	2 (0.017)	99 (0.825)	0	1 (0.008)	1 (0.008)	13 (0.108)	120
**Cameroon**	0	0	3 (0.002)	1587 (0.847)	0	2 (0.001)	59 (0.032)	223 (0.119)	1874
**Chad**	3 (0.01)	0	18 (0.058)	288 (0.926)	0	0	2 (0.006)	0	311
**Congo**	1 (0.014)	1 (0.014)	2 (0.027)	64 (0.865)	0	0	6 (0.081)	0	74
**Djibouti**	4 (0.065)	0	14 (0.226)	30 (0.484)	0	1 (0.016)	0	13 (0.21)	62
**Egypt**	0	0	14 (0.061)	175 (0.761)	0	0	0	41 (0.178)	230
**Ethiopia**	48 (0.019)	3 (0.001)	591 (0.238)	1582 (0.638)	162 (0.065)	8 (0.003)	37 (0.015)	47 (0.019)	2478
**Gabon**	12 (0.038)	12 (0.038)	0	247 (0.777)	0	0	10 (0.031)	37 (0.116)	318
**Gambia**	6 (0.017)	10 (0.028)	6 (0.017)	188 (0.524)	0	0	138 (0.384)	11 (0.031)	359
**Ghana**	53 (0.014)	116 (0.031)	37 (0.01)	2748 (0.732)	0	23 (0.006)	778 (0.207)	1	3756
**Guinea-Bissau**	30 (0.074)	7 (0.017)	3 (0.007)	164 (0.403)	0	0	195 (0.479)	8 (0.02)	407
**Kenya**	16 (0.03)	66 (0.123)	129 (0.241)	278 (0.519)	0	0	0	47 (0.088)	536
**Madagascar**	60 (0.134)	45 (0.1)	45 (0.1)	286 (0.637)	0	3 (0.007)	0	10 (0.022)	449
**Malawi**	807 (0.159)	222 (0.044)	615 (0.122)	3417 (0.675)	0	0	0	0	5061
**Mali**	6 (0.048)	1 (0.008)	2 (0.016)	84 (0.667)	0	1 (0.008)	32 (0.254)	0	126
**Morocco**	0	5 (0.007)	0	694 (0.918)	0	0	0	57 (0.076)	755
**Mozambique**	132 (0.297)	31 (0.07)	11 (0.025)	255 (0.573)	0	0	0	16 (0.036)	445
**Nigeria**	7 (0.039)	0	0	152 (0.844)	0	0	21 (0.117)	0	180
**South Africa**	5 (0.007)	115 (0.166)	15 (0.022)	501 (0.722)	0	0	0	58 (0.084)	694
**Sudan**	0	7 (0.03)	130 (0.56)	32 (0.138)	0	0	0	63 (0.272)	232

**Tanzania**	201 (0.143)	64 (0.046)	501 (0.357)	565 (0.403)	0	0	0	72 (0.051)	1403
**Tunisia**	1 (0.003)	0	0	374 (0.989)	0	0	0	3 (0.008)	378
**Uganda**	12 (0.013)	9 (0.01)	82 (0.089)	656 (0.711)	0	0	0	164 (0.178)	923
**Zambia**	2 (0.007)	0	2 (0.007)	174 (0.637)	0	0	0	95 (0.348)	273
**Zimbabwe**	0	0	0	80 (0.69)	0	0	0	36 (0.31)	116
Americas	*16502 (0.151)*	*17731 (0.163)*	*3676 (0.034)*	*68790 (0.631)*	*0*	*827 (0.008)*	*352 (0.003)*	*1215 (0.011)*	*109093*
**Brazil**	110 (0.041)	7 (0.003)	8 (0.003)	2423 (0.896)	0	0	1	154 (0.057)	2703
**Canada**	1109 (0.211)	982 (0.187)	658 (0.125)	2149 (0.409)	0	25 (0.005)	5 (0.001)	327 (0.062)	5255
**Chile**	0	4 (0.008)	3 (0.006)	487 (0.984)	0	0	1 (0.002)	0	495
**Colombia**	9 (0.012)	24 (0.032)	1 (0.001)	570 (0.769)	0	1 (0.001)	0	136 (0.184)	741
**Cuba**	0	106 (0.194)	0	367 (0.671)	0	0	0	74 (0.135)	547
**Greenland**	0	0	0	182 (1)	0	0	0	0	182
**Honduras**	0	1 (0.005)	0	194 (0.942)	0	0	0	11 (0.053)	206
**Mexico**	185 (0.094)	26 (0.013)	19 (0.01)	1490 (0.756)	0	5 (0.003)	14 (0.007)	233 (0.118)	1972
**Panama**	60 (0.143)	20 (0.048)	2 (0.005)	321 (0.764)	0	0	17 (0.04)	0	420
**Paraguay**	0	1 (0.006)	2 (0.012)	170 (0.983)	0	0	0	0	173
**Peru**	0	355 (0.121)	0	2344 (0.796)	0	0	0	245 (0.083)	2944
**Trinidad and Tobago**	4 (0.03)	6 (0.045)	1 (0.008)	107 (0.811)	0	0	0	14 (0.106)	132
**United States**	15025 (0.162)	16199 (0.175)	2982 (0.032)	57374 (0.619)	0	796 (0.009)	312 (0.003)	0	92688
**Venezuela**	0	0	0	612 (0.964)	0	0	2 (0.003)	21 (0.033)	635
East Asia	*2065 (0.094)*	*12733 (0.582)*	*534 (0.024)*	*3770 (0.172)*	*0*	*7*	*2*	*2768 (0.127)*	*21879*
**China**	38 (0.003)	9141 (0.722)	93 (0.007)	2103 (0.166)	0	0	0	1277 (0.101)	12652
**Indonesia**	204 (0.116)	504 (0.287)	3 (0.002)	552 (0.314)	0	1 (0.001)	1 (0.001)	492 (0.28)	1757
**Japan**	13 (0.078)	110 (0.663)	2 (0.012)	41 (0.247)	0	0	0	0	166
**Korea, Republic of**	10 (0.033)	278 (0.914)	0	15 (0.049)	0	1 (0.003)	0	0	304
**Malaysia**	125 (0.568)	63 (0.286)	3 (0.014)	24 (0.109)	0	1 (0.005)	0	4 (0.018)	220
**Mongolia**	0	65 (0.58)	0	47 (0.42)	0	0	0	0	112
**Myanmar**	149 (0.481)	100 (0.323)	15 (0.048)	32 (0.103)	0	0	1 (0.003)	13 (0.042)	310
**Philippines**	110 (0.973)	1 (0.009)	0	2 (0.018)	0	0	0	0	113

**Singapore**	9 (0.17)	21 (0.396)	0	11 (0.208)	0	0	0	12 (0.226)	53
**Taiwan**	418 (0.188)	966 (0.434)	0	642 (0.288)	0	4 (0.002)	0	194 (0.087)	2224
**Viet Nam**	989 (0.249)	1484 (0.374)	418 (0.105)	301 (0.076)	0	0	0	776 (0.196)	3968
Europe	*3412 (0.108)*	*2048 (0.065)*	*4803 (0.152)*	*17135 (0.544)*	*0*	*343 (0.011)*	*303 (0.01)*	*3455 (0.11)*	*31499*
**Belgium**	19 (0.021)	37 (0.041)	51 (0.056)	773 (0.849)	0	14 (0.015)	16 (0.018)	0	910
**Bulgaria**	1 (0.009)	0	0	101 (0.886)	0	0	0	12 (0.105)	114
**Croatia**	1 (0.001)	2 (0.001)	0	1059 (0.667)	0	0	1 (0.001)	524 (0.33)	1587
**Czech Republic**	0	7 (0.033)	0	109 (0.522)	0	0	0	93 (0.445)	209
**Estonia**	0	357 (0.391)	0	421 (0.462)	0	0	0	134 (0.147)	912
**Finland**	119 (0.083)	80 (0.056)	47 (0.033)	938 (0.657)	0	1 (0.001)	3 (0.002)	240 (0.168)	1428
**France**	94 (0.033)	90 (0.032)	31 (0.011)	2235 (0.786)	0	96 (0.034)	39 (0.014)	257 (0.09)	2842
**Ireland**	54 (0.075)	65 (0.09)	51 (0.071)	423 (0.588)	0	6 (0.008)	0	120 (0.167)	719
**Italy**	33 (0.025)	47 (0.036)	35 (0.027)	997 (0.756)	0	31 (0.024)	15 (0.011)	161 (0.122)	1319
**Netherlands**	384 (0.102)	274 (0.073)	328 (0.087)	2621 (0.694)	0	62 (0.016)	59 (0.016)	48 (0.013)	3776
**Portugal**	20 (0.023)	101 (0.114)	2 (0.002)	660 (0.747)	0	0	10 (0.011)	91 (0.103)	884
**Spain**	4 (0.013)	0	2 (0.006)	310 (0.975)	0	1 (0.003)	1 (0.003)	0	318
**Sweden**	53 (0.093)	38 (0.067)	49 (0.086)	267 (0.469)	0	2 (0.004)	0	160 (0.281)	569
**Ukraine**	0	32 (0.327)	0	30 (0.306)	0	0	0	36 (0.367)	98
**United Kingdom**	2630 (0.166)	918 (0.058)	4207 (0.266)	6191 (0.391)	0	130 (0.008)	159 (0.01)	1579 (0.1)	15814
Oceania	*686 (0.198)*	*913 (0.264)*	*401 (0.116)*	*707 (0.204)*	*0*	*3 (0.001)*	*1*	*752 (0.217)*	*3463*
**Australia**	569 (0.22)	676 (0.262)	315 (0.122)	290 (0.112)	0	0	0	735 (0.284)	2585
**Kiribati**	1 (0.014)	36 (0.507)	0	25 (0.352)	0	0	0	9 (0.127)	71
**New Zealand**	110 (0.226)	95 (0.195)	86 (0.177)	184 (0.378)	0	3 (0.006)	1 (0.002)	8 (0.016)	487
**Papua New Guinea**	6 (0.019)	106 (0.331)	0	208 (0.65)	0	0	0	0	320
West Asia	*4646 (0.106)*	*4659 (0.107)*	*13470 (0.308)*	*15934 (0.365)*	*0*	*226 (0.005)*	*1*	*4737 (0.108)*	*43673*
**Bangladesh**	105 (0.432)	39 (0.16)	35 (0.144)	33 (0.136)	0	0	0	31 (0.128)	243
**Georgia**	0	220 (0.323)	2 (0.003)	418 (0.613)	0	1 (0.001)	0	41 (0.06)	682

**India**	3498 (0.483)	370 (0.051)	1358 (0.188)	548 (0.076)	0	0	0	1466 (0.202)	7240
**Iran, Islamic Republic of**	527 (0.019)	2230 (0.079)	10286 (0.366)	12491 (0.445)	0	210 (0.007)	1	2347 (0.084)	28092
**Iraq**	1 (0.005)	0	69 (0.377)	113 (0.617)	0	0	0	0	183
**Israel**	39 (0.057)	101 (0.148)	140 (0.205)	294 (0.43)	0	0	0	109 (0.16)	683
**Nepal**	0	0	64 (0.364)	69 (0.392)	0	0	0	43 (0.244)	176
**Oman**	50 (0.234)	13 (0.061)	85 (0.397)	38 (0.178)	0	0	0	28 (0.131)	214
**Pakistan**	178 (0.103)	52 (0.03)	1085 (0.63)	139 (0.081)	0	0	0	269 (0.156)	1723
**Russian Federation**	3 (0.001)	1553 (0.574)	7 (0.003)	1083 (0.401)	0	2 (0.001)	0	56 (0.021)	2704
**Saudi Arabia**	215 (0.143)	67 (0.045)	339 (0.225)	587 (0.39)	0	13 (0.009)	0	284 (0.189)	1505
**Sri Lanka**	30 (0.3)	14 (0.14)	0	13 (0.13)	0	0	0	43 (0.43)	100
**Turkey**	0	0	0	108 (0.844)	0	0	0	20 (0.156)	128

55

Table S4. Summary of studies included in random effects (RE) meta-analysis of genetic clustering. These studies are included in the RE meta-analyses in Figure S4 and Table 1. Raw data corresponding to these studies can be found in Additional file 4. Dashes indicate that the study design or confounder variable was not reported.

PubMed ID	Location	Year (mid-point)	Collection period (months)	Sampling method	Genotyping method	Sample size	HIV (proportion)	Age (mean)	Previous TB (proportion)	Male (proportion)	Immigrants (proportion)
**Africa**											
16970826	Dar es Salaam, Tanzania	2005	2	All cases	Spoligotyping	145	-	-	-	-	-
19193842	The Gambia	2007	-	All new patients	Spoligotyping	359	0.08	29.00	0.00	0.69	-
22414165	Jimma Zone, Ethiopia	2009	2	All new patients	Spoligotyping	17	0.06	41.00	0.50	0.39	-
23496968	Northwest Ethiopia	2009	3	All cases	Spoligotyping	244	0.25	31.60	0.17	0.58	0.10
24734230	Afar Region, Ethiopia	2010	6	All new patients	Spoligotyping	105	0.39	29.00	0.27	0.66	-
26285026	Morocco	2011	24	Cluster	Spoligotyping	168	-	49.50	0.01	0.74	-
26491657	Bahir Dar, Ethiopia	2013	16	All cases	Spoligotyping	170	-	-	0.15	0.48	-
28230095	Luanda District, Angola	2014	12	All cases	Spoligotyping	88	0.15	-	0.11	-	-
26927993	Anambra State, Nigeria	2010	24	All cases	MLVA	180	0.19	35.00	0.16	0.61	-
27149626	Tanzania	2011	24	All cases	Spoligotyping	53	0.10	25.10	-	0.53	-
27506391	Ghana	2013	18	All new patients	Spoligotyping	1205	0.13	39.00	-	0.69	-
**West Asia**											
19108722	Pakistan	2004	36	Cluster	Spoligotyping	675	-	-	-	0.55	-
21394425	Murmansk, Russia	2004	24	All cases	Spoligotyping	387	-	-	0.64	-	-
22844457	Psov, Russia	2009	6	Simple random	Spoligotyping	90	0.00	-	0.28	0.66	-
26679959	Republic of Karelia, Russia	2014	18	All new patients	MLVA	78	-	43.40	0.88	0.72	0.00
26786944	Duhok, Iraq	2009	12	All cases	MLVA	49	-	-	-	-	-
**East Asia**											
19036933	Yangon Division, Myanmar	2002	9	All cases	Spoligotyping	310	-	36.60	0.44	0.70	-
22912700	Tien Giang Province, Vietnam	2005	48	All new patients	MLVA	2207	-	-	0.10	0.75	-
25629610	Kaohsiung, Taiwan	2004	96	Simple random	Both	421	-	-	-	-	-
28333978	China	2007	12	Cluster	Spoligotyping	3133	-	-	0.25	-	-

**Europe**											
21439097	Kharviv, Ukraine	2004	3	All cases	MLVA	98	-	-	0.63	-	-
23658260	The Netherlands	2006	60	All cases	MLVA	3776	-	-	0.06	0.58	-
28222189	Brussels, Belgium	2012	36	All cases	MLVA	910	0.08	38.40	0.07	0.64	0.76
**Americas**											
22377473	United States	2007	48	All cases	MLVA	36458	0.91	-	0.05	0.66	-
22982156	San Diego, USA	2007	36	All cases	Both	832	0.12	-	0.06	0.65	0.78
24098337	Espirito Santo, Brazil	2003	108	All new patients	Spoligotyping	506	0.13	35.90	-	0.70	-
25671320	Santiago, Chile	2011	60	All cases	MLVA	87	-	-	-	0.61	0.12
25809979	San Juan de Lurigancho, Lima, Peru	2011	22	All new patients	Both	791	-	-	0.00	0.61	-
27518286	Chile	2012	12	All cases	MLVA	408	-	-	0.00	0.66	-
28317799	Veracruz, Mexico	2015	12	All cases	Spoligotyping	79	0.00	42.00	0.00	0.65	-

Supplementary figures

Figure S1. Human TB molecular epidemiology studies 1990-2017. Number of studies that met the inclusion criteria for this review by location/year in each country. The systematic review included human TB molecular epidemiology studies that performed genotyping methods that could be converted to a common classification system based on phylogenetic lineages (Table S1, Table S2). Studies that used unclear or convenience sampling methods, monitored bacterial or human sub-populations, and/or only reported results for one phylogenetic lineage were excluded.

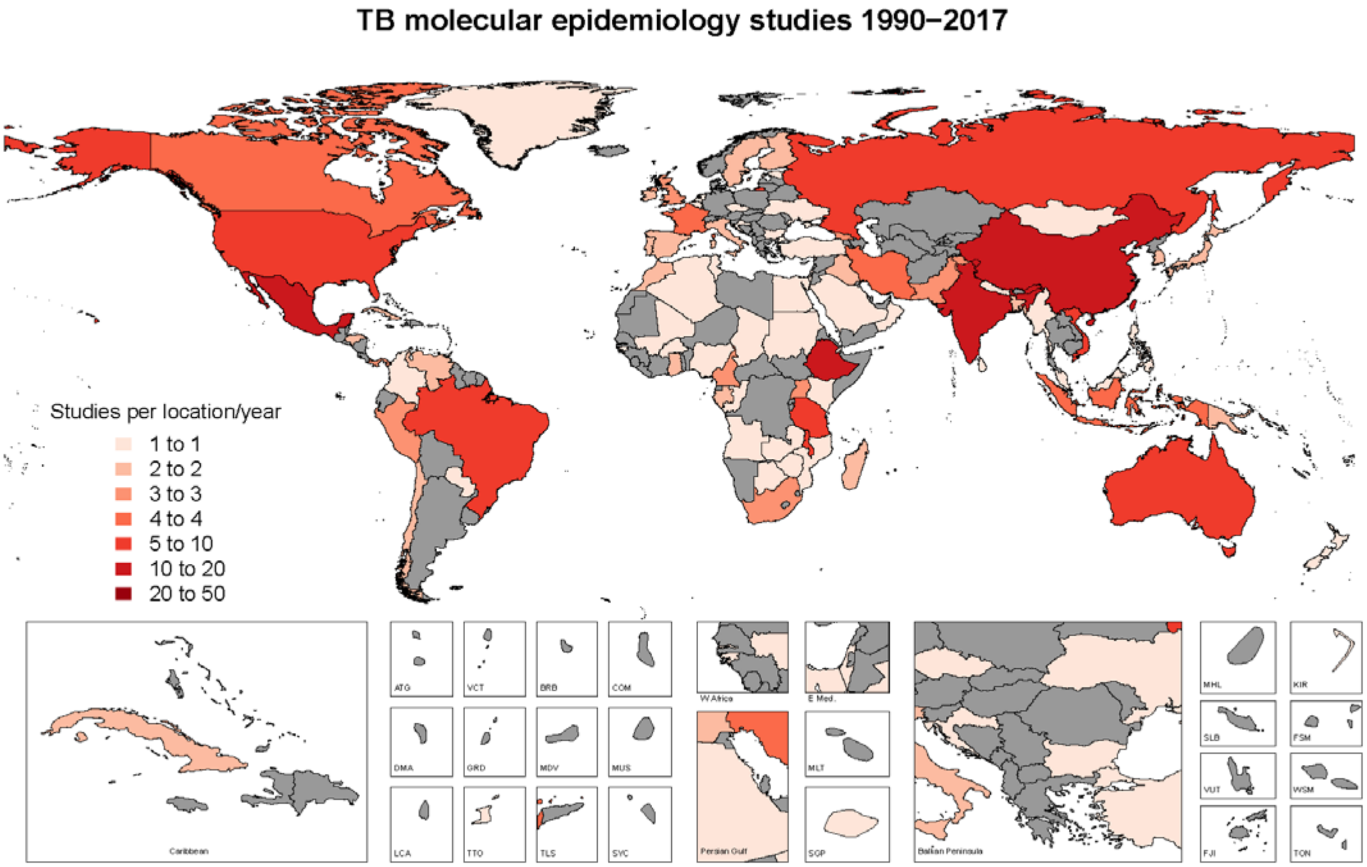

Figure S2. Genetic diversity and global distribution of MTBC phylogenetic lineages. Stacked bar charts of MTBC global genotype distribution based on a systematic review of TB molecular epidemiology studies, corresponding to the results in Figure 2, Figure 3, and Table S3. Results are shown by country and the following regions: Africa, Oceania, West Asia, North and South America, East Asia, and Europe. Countries are indicated by ISO Alpha-3 codes.

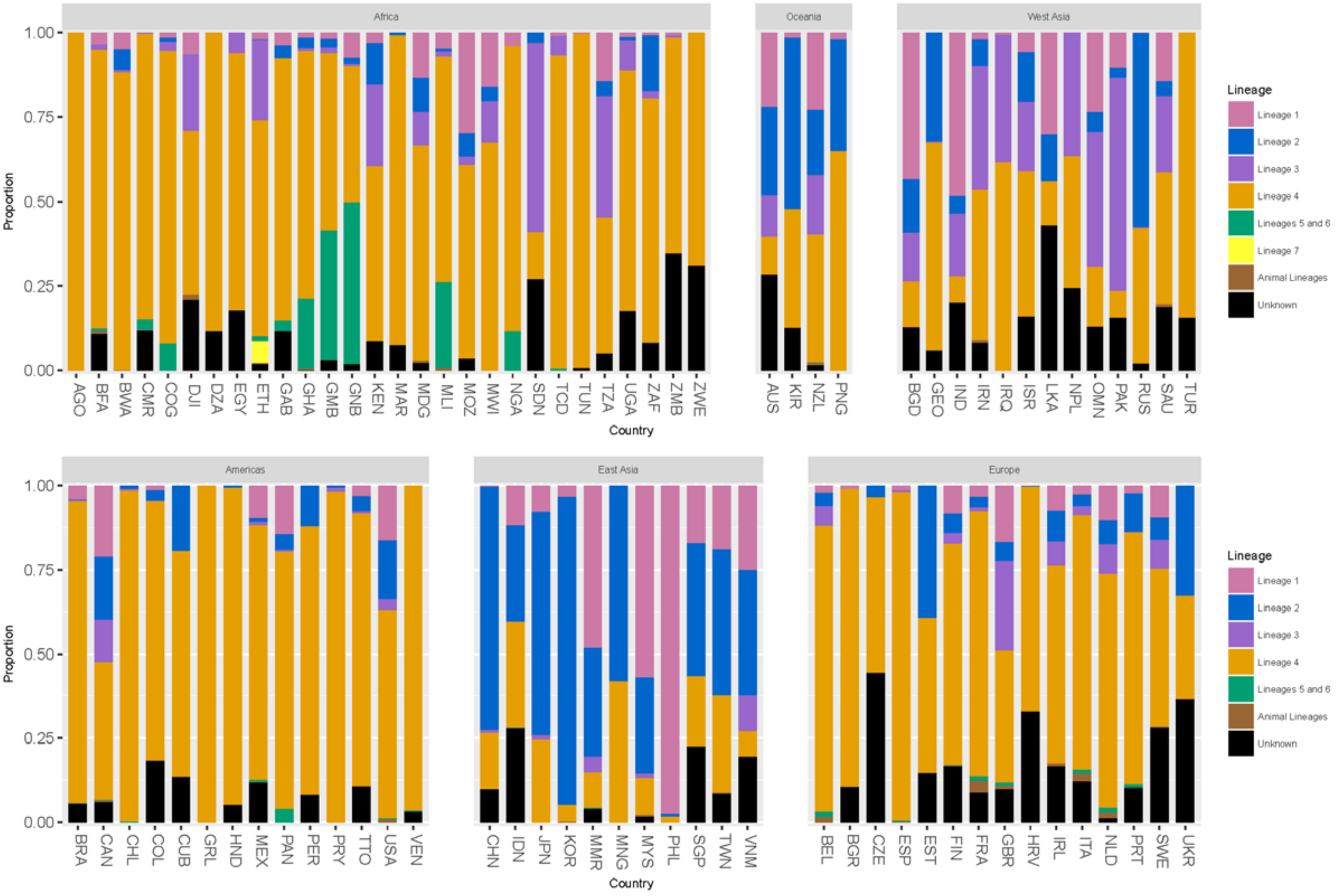

Figure S3. Global distribution and genetic diversity and of MTBC phylogenetic lineages over time. MTBC global genotype distribution by country from (A) 1990 to 1999, (B) 2000 to 2004, (C) 2005 to 2009, and (D) 2010 to 2017. Indo-Oceanic lineage 1 is shown in pink, lineage 2 is shown in blue, East African-Indian lineage 3 is shown in purple, Euro-American lineage 4 is shown in orange, West African lineages 5 and 6 are shown in green, and Ethiopian lineage 7 is shown in yellow. “Unknown” represents strain types that were not identified by authors either due to low frequency or unknown genetic patterns. If multiple studies were available in a country, strain counts were summed across all studies within the given time period to get final proportions and sample sizes. The radius of each pie is proportional to the number of isolates collected in each country. The range of sample sizes that correspond to the radii of the pies are shown in the legend in grey pies. The example pies shown represent the minimum, mid-point, and maximum sample sizes.

Figure S3A. MTBC global genotype distribution by country 1990-1999.

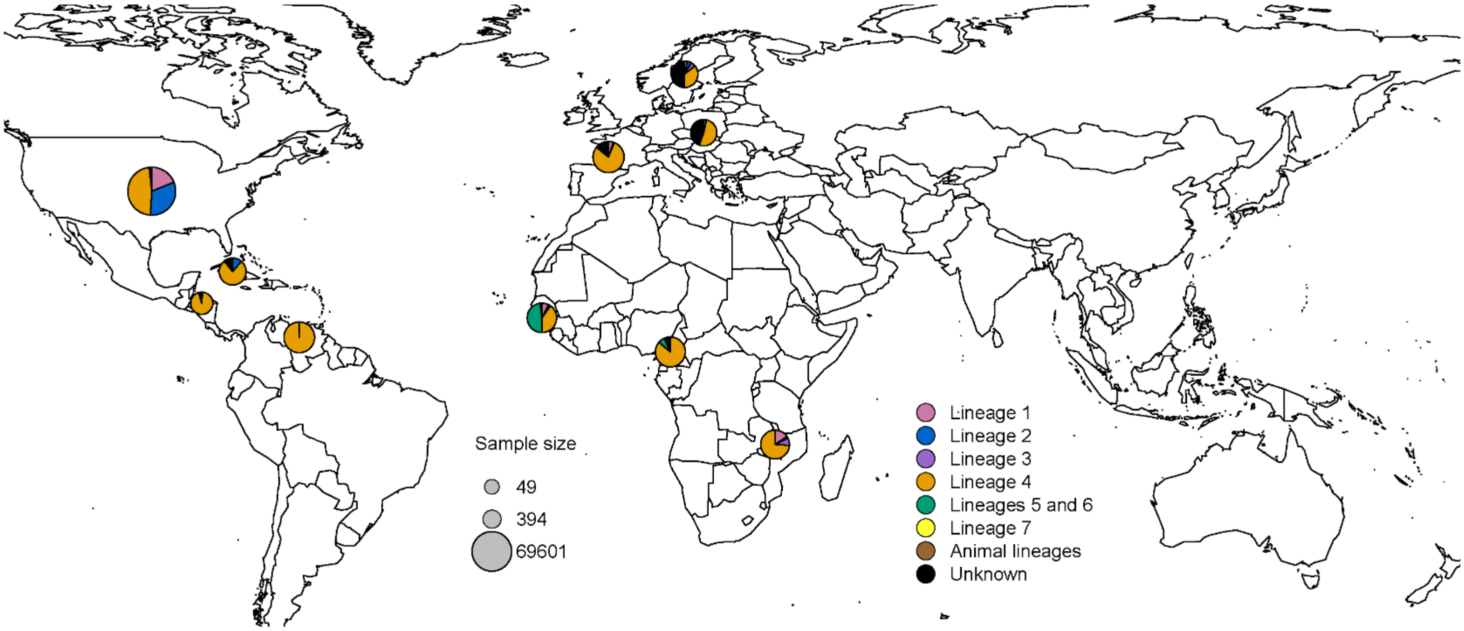

Figure S3B. MTBC global genotype distribution by country 2000-2004.

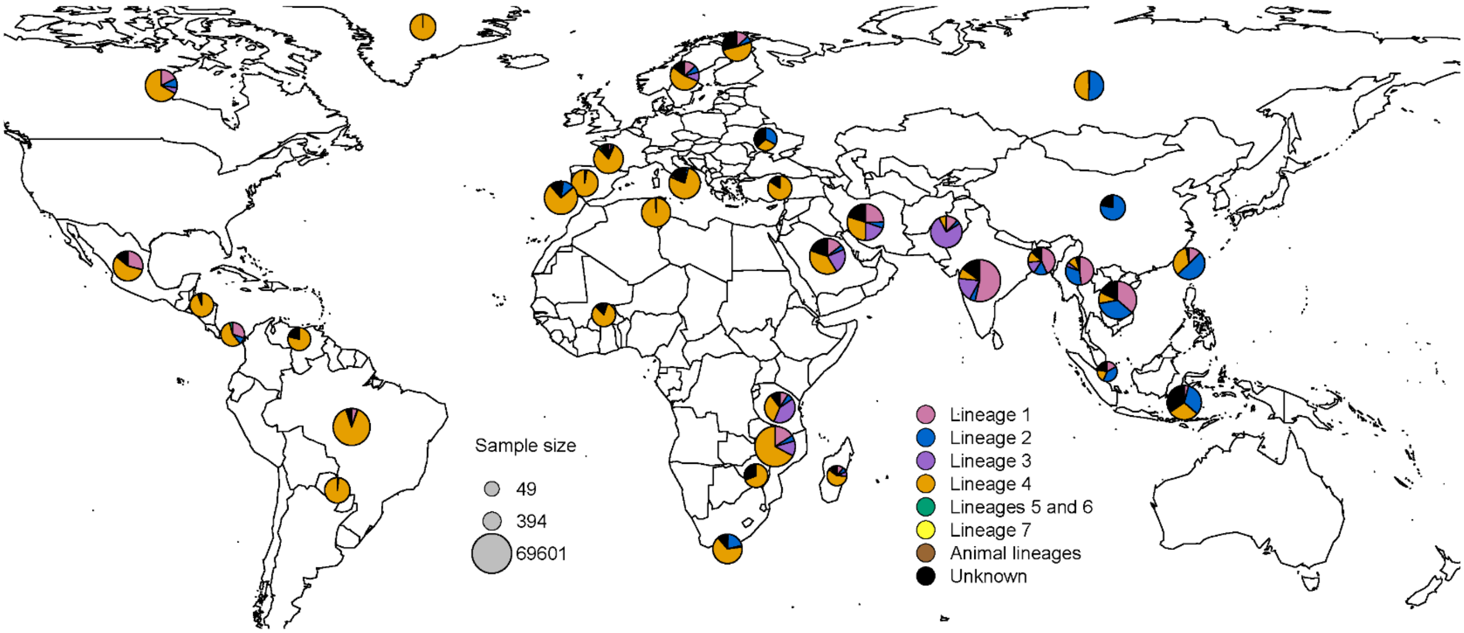

Figure S3C. MTBC global genotype distribution by country 2005-2009.

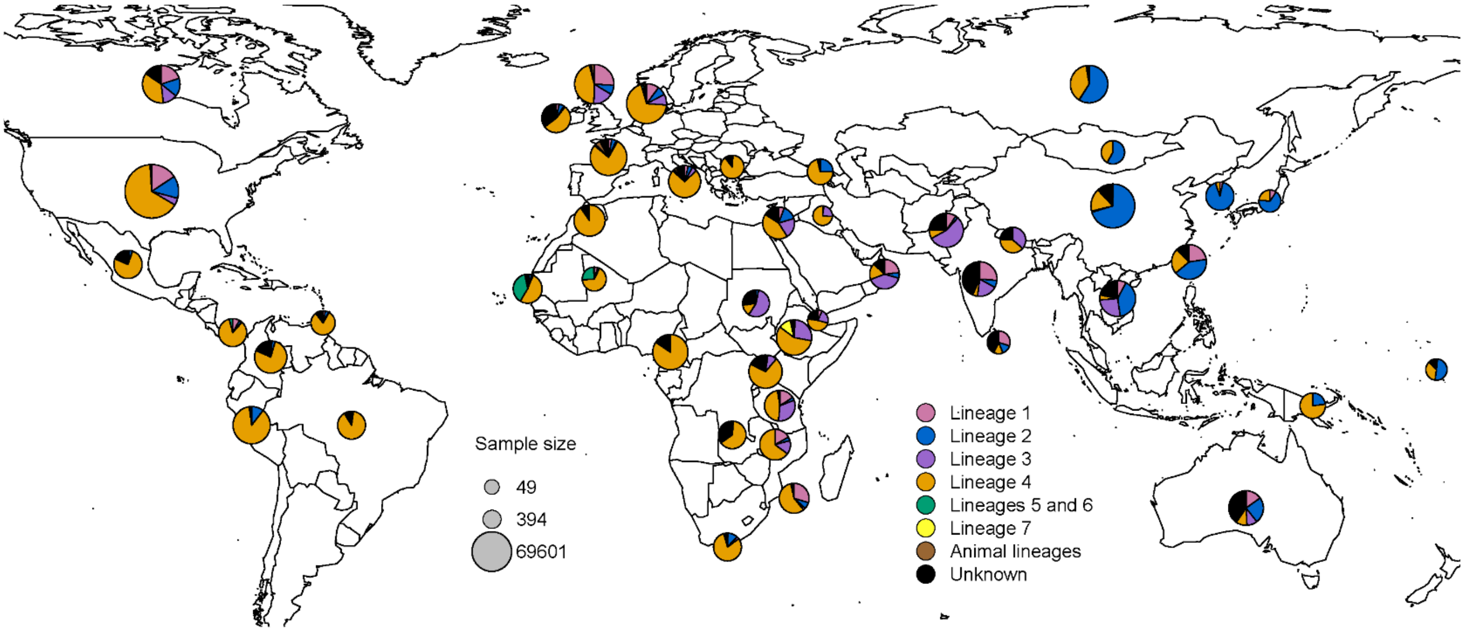

Figure S3D. MTBC global genotype distribution by country 2010-2017.

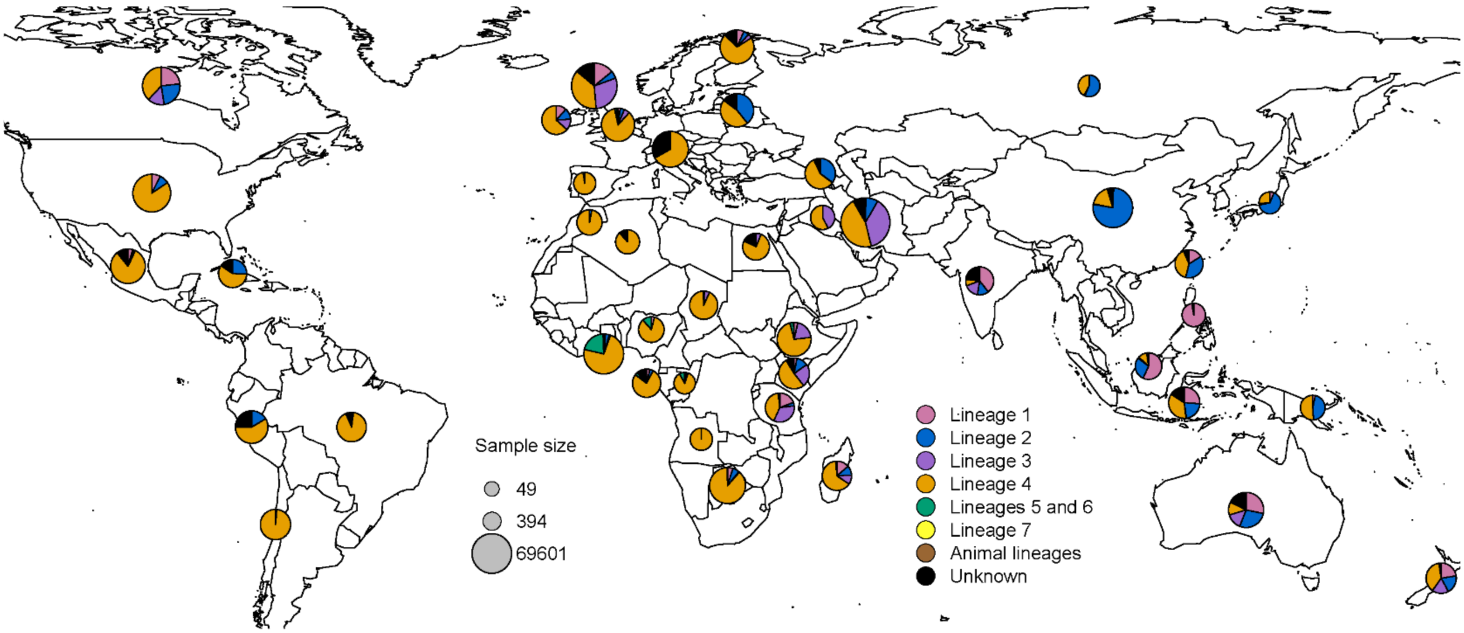

110

Figure S4. Random effects (RE) meta-analyses of the relative risk (RR) of transmission chains associated with MTBC lineages. RE meta-analysis of the RR of transmission chains associated with lineage 1 (A), lineage 2 Beijing (B), lineage 3 (C), lineage 5 and 6 (D), animal (E), and unknown (F) strains compared with lineage 4 strains as the reference group (A-F). Transmission chains in this analysis are defined as identification of two or more MTBC isolates with identical genetic patterns in the same study location and time period. "Cluster" indicates part of a transmission chain, and "unique" indicates not part of a transmission chain. We performed the analysis across all studies that reported transmission clusters by MTBC genotype that we identified in the systematic review, as well as within the regions West Asia, East Asia, Europe and Americas, and Africa. We excluded studies that identified less than two isolates of lineage 2 Beijing (A), lineage 1 (B), lineage 3 (C), lineage 5 and 6 (D), animal (E), or unknown (F) strains and studies that identified less than two isolates of lineage 4 strains (A-D). In addition, we added ½ a value to studies that identified zero isolates in the "cluster" category. RE meta-analysis was performed using the RELM method in R package 'metafor'. Q values with corresponding degrees of freedom (df), p, and I^2 values are shown across all studies and within each region. RRs with corresponding confidence intervals (CI) are also shown across all studies and within each region. These analyses correspond to the results shown in Table 1.

Figure S4A. RR of transmission chains for lineage 1 strains.

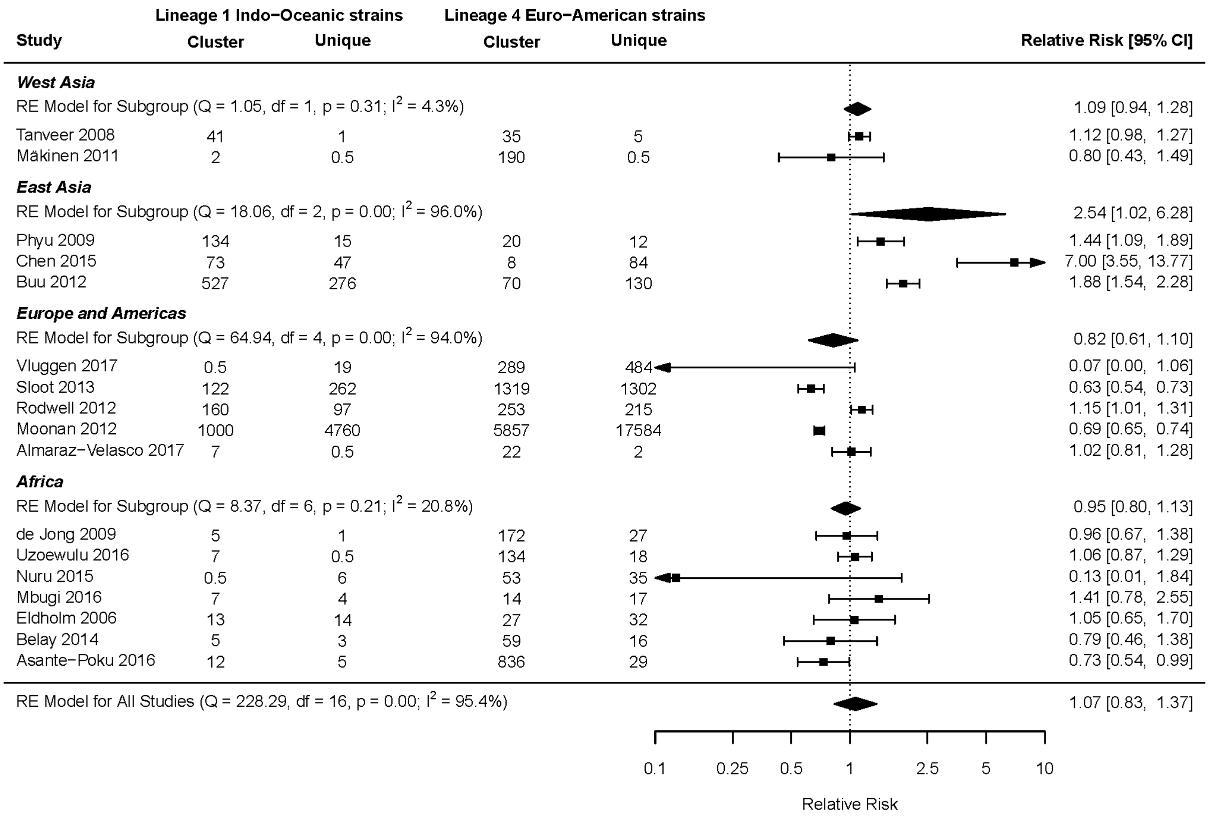

Figure S4B. RR of transmission chains for lineage 2 Beijing strains.

Study	Lineage 2 East Asian strains Cluster	Lineage 2 East Asian strains Unique	Lineage 4 Euro−American strains Cluster	Lineage 4 Euro−American strains Unique	Relative Risk [95% CI]
West Asia					
RE Model for Subgroup (Q = 15.28, df = 3, p = 0.00; I^2 = 88.1%)					1.12 [0.97, 1.29]
Tanveer 2008	25	0.5	35	5	1.12 [0.98, 1.28]
Mäkinen 2011	193	0.5	190	0.5	1.00 [0.99, 1.01]
Mokrousov 2015	42	2	20	13	1.58 [1.19, 2.09]
Mokrousov 2012	40	0.5	43	4	1.08 [0.98, 1.19]
East Asia					
RE Model for Subgroup (Q = 52.04, df = 3, p = 0.00; I^2 = 93.5%)					1.90 [1.14, 3.17]
Zhou 2017	1061	1147	121	565	2.72 [2.30, 3.22]
Phyu 2009	98	2	20	12	1.57 [1.20, 2.05]
Chen 2015	53	123	8	84	3.46 [1.72, 6.97]
Buu 2012	292	494	70	130	1.06 [0.86, 1.31]
Europe and Americas					
RE Model for Subgroup (Q = 28.59, df = 7, p = 0.00; I^2 = 82.9%)					1.05 [0.88, 1.24]
Vluggen 2017	12	25	289	484	0.87 [0.54, 1.39]
Sloot 2013	126	148	1319	1302	0.91 [0.80, 1.04]
Rodwell 2012	58	21	253	215	1.36 [1.16, 1.59]
Moonan 2012	1259	3665	5857	17584	1.02 [0.97, 1.08]
Lagos 2016	2	0.5	323	81	1.00 [0.54, 1.86]
Dymova 2011	18	14	9	21	1.88 [1.00, 3.51]
Barletta 2015	77	53	336	127	0.82 [0.70, 0.95]
Balcells 2015	2	0.5	47	36	1.41 [0.74, 2.70]
Africa					
RE Model for Subgroup (Q = 16.67, df = 3, p = 0.00; I^2 = 89.1%)					1.21 [0.90, 1.63]
de Jong 2009	9	1	172	27	1.04 [0.84, 1.29]
Eldholm 2006	7	0.5	27	32	2.04 [1.46, 2.86]
Bouklata 2015	5	0.5	134	29	1.11 [0.84, 1.45]
Asante−Poku 2016	38	1	836	29	1.01 [0.96, 1.06]
RE Model for All Studies (Q = 220.56, df = 19, p = 0.00; I^2 = 97.8%)					1.24 [1.07, 1.45]

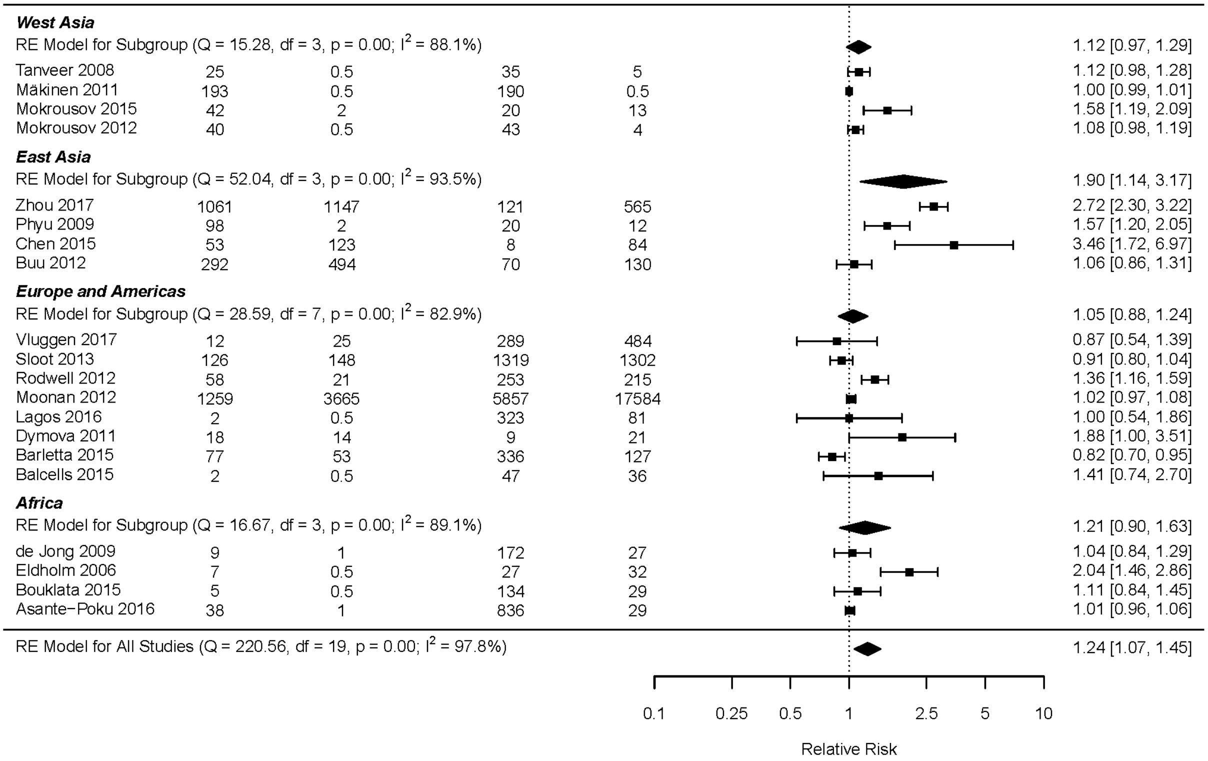

Figure S4C. RR of transmission chains for lineage 3 strains.

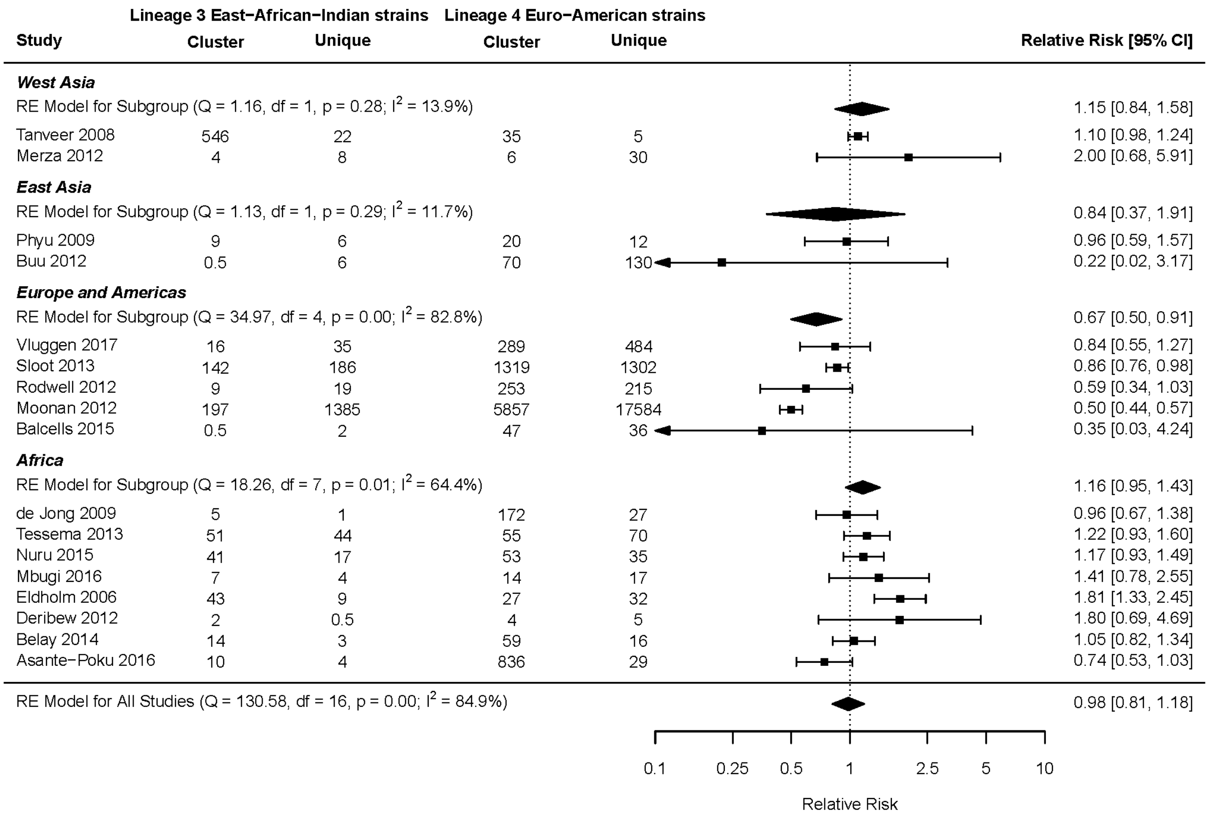

Figure S4D. RR of transmission chains for lineage 5 and 6 strains.

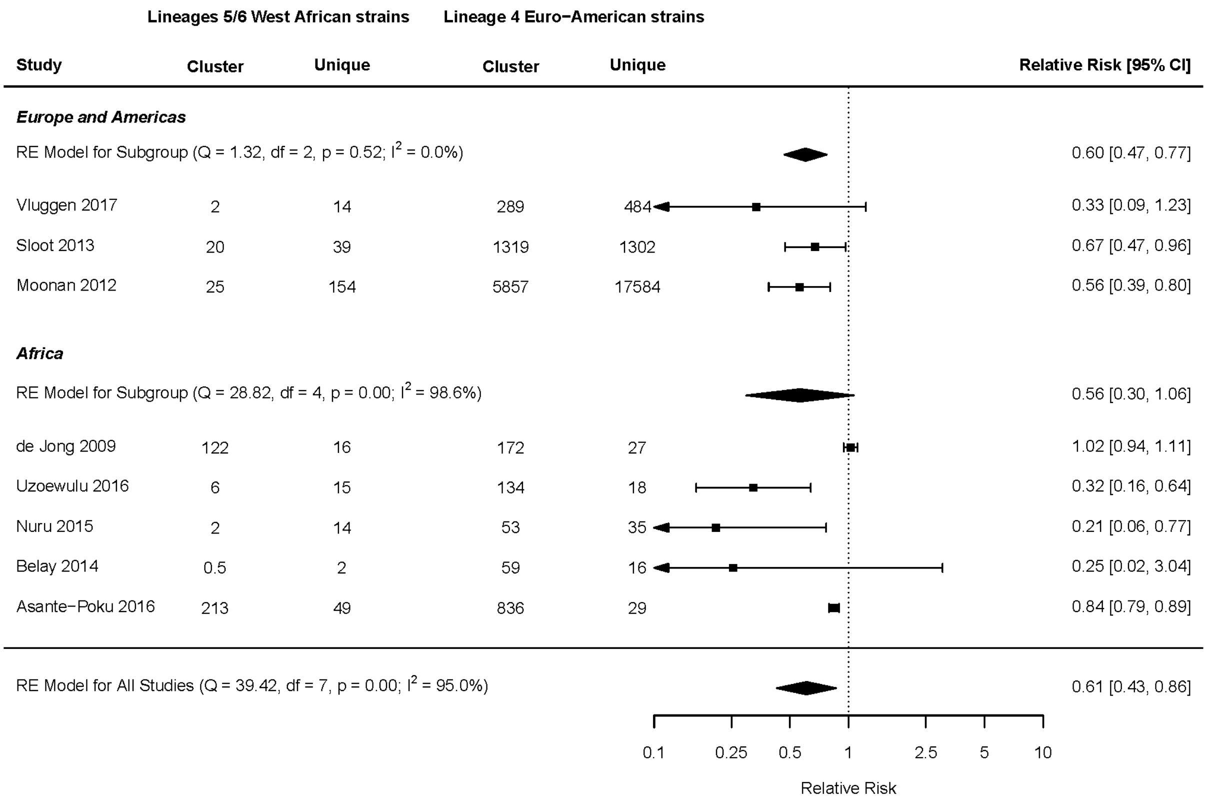

Figure S4E. RR of transmission chains for animal strains.

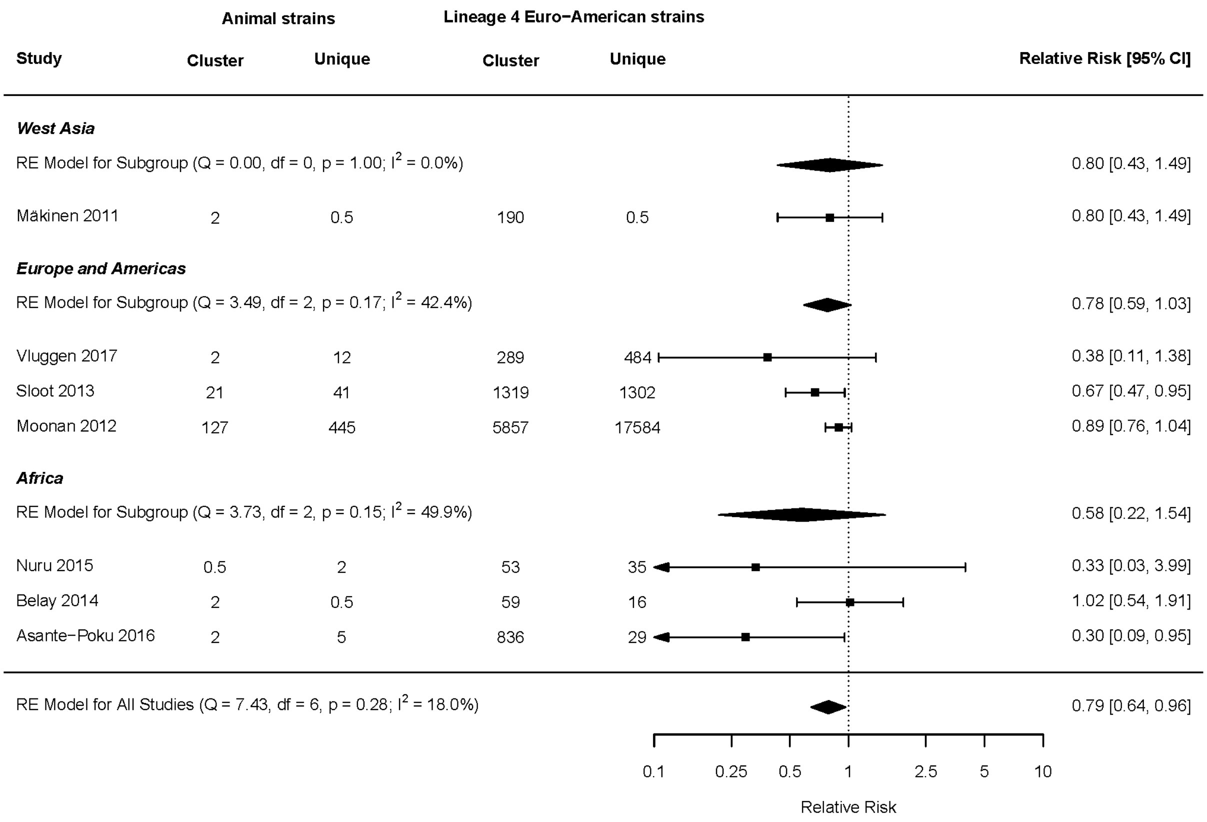

Figure S4F. RR of transmission chains for unknown strains.

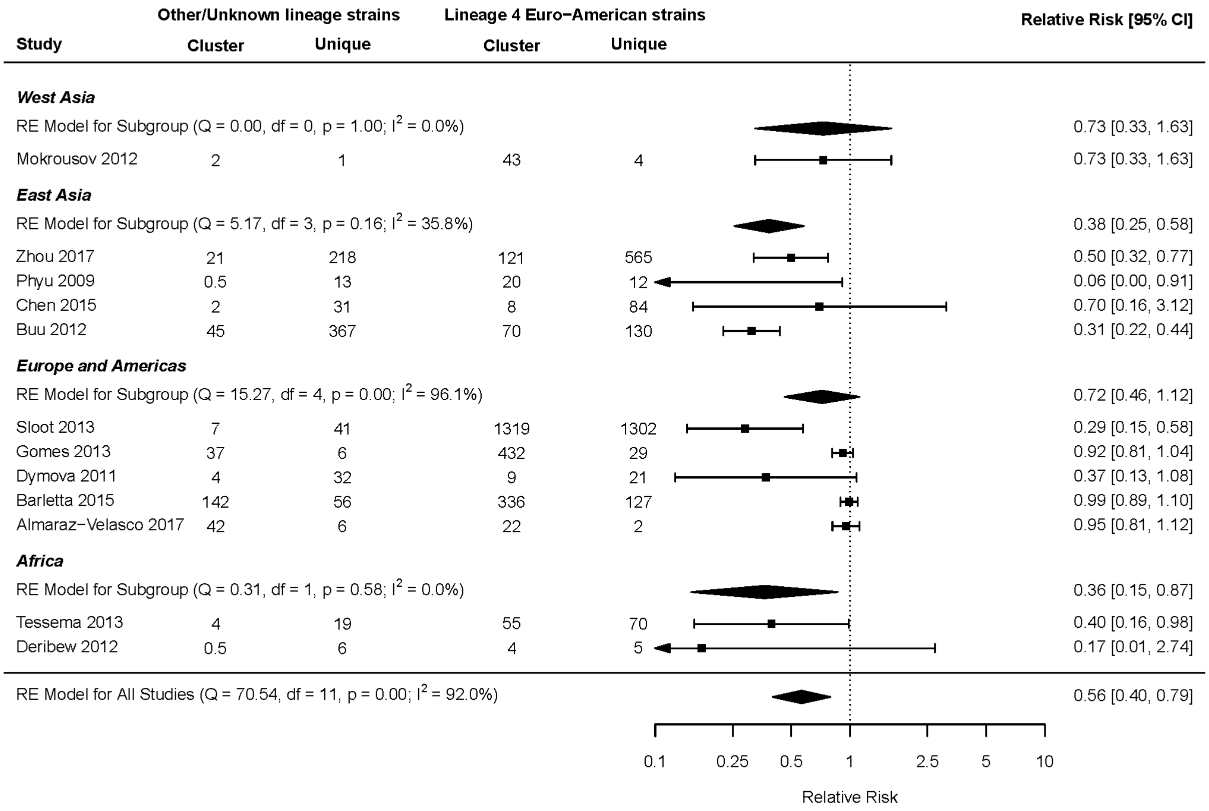

Supplement: Supplementary file 1 — Supplementary appendix. Document containing complete description of literature search strings and dates searched, as well as Tables S1-S4 and Figure S1-S4. (PDF 1440 kb) [file 12916_2018_1180_MOESM1_ESM.pdf]
